# An Optimized Protein Extraction Method for Gel-Free Proteomic Analysis of *Opuntia Ficus-Indica*

**DOI:** 10.3390/plants10010115

**Published:** 2021-01-08

**Authors:** Akiko Hashiguchi, Hisateru Yamaguchi, Keisuke Hitachi, Kazuo Watanabe

**Affiliations:** 1 Faculty of Medicine, University of Tsukuba, Tsukuba 305-8575, Japan; hashiguchi.akiko.ge@u.tsukuba.ac.jp; 2 School of Nursing and Medical Care, Yokkaichi Nursing and Medical Care University, Yokkaichi 512-8045, Japan; h-yamaguchi@y-nm.ac.jp; 3 Institute for Comprehensive Medical Science, Fujita Health University, Toyoake 470-1192, Japan; hkeisuke@fujita-hu.ac.jp; 4 Tsukuba-Plant Innovation Research Center, University of Tsukuba, Tsukuba 305-8577, Japan; 5 Faculty of Life and Environmental Sciences, University of Tsukuba, Tsukuba 305-8577, Japan

**Keywords:** *Opuntia*, succulent, protein extraction, gel-free proteomic analysis, abiotic stress, environmental stress

## Abstract

*Opuntia* spp. is an economically important vegetable crop with high stress-tolerance and health benefits. However, proteomic analysis of the plant has been difficult due to the composition of its succulent cladodes; the abundant polysaccharides interfere with protein extraction. To facilitate proteomic analysis of this plant, we present a rapid and simple protein extraction method for *Opuntia ficus-indica* (L.) Miller. The optimized method produced highly reproducible protein patterns and was compatible with a gel-free quantitative workflow without the need for additional purification. We successfully analyzed the cladode mesocarp and exocarp tissues, resulting in the identification of 319 proteins. In addition, we used this method to examine the relative changes in the *Opuntia* proteome in response to salt stress to determine whether physiological changes could be captured. Qualified observations were obtained, revealing that salt stress increased phosphoenolpyruvate carboxylase abundance and decreased ribulose-bisphosphate carboxylase in young *O. ficus-indica* plants. These findings suggest that Crassulacean acid metabolism is promoted under salinity. This study highlights the efficacy of our optimized protein extraction method for elucidating the metabolic adaptations of *Opuntia* using gel-free proteomic analysis.

## 1. Introduction

The *Opuntia* is a large genus of succulents (family: Cactaceae) comprising Crassulacean acid metabolism (CAM) plants native to the New World. *Opuntia* spp. are grown as vegetable and fruit crops in America, Africa, and the Mediterranean [1]. This genus has become an important alternative crop in the context of climate change, due to the high water-use efficiency and heat/drought tolerance of *Opuntia* spp. [2]. The undemanding growth of *Opuntia* spp. under harsh conditions is supported by intricate growth–defense tradeoffs, as demonstrated in plants attacked by carmine cochineal pests [3]. In addition, *Opuntia* spp. accumulate various secondary metabolites with health-promoting effects. Consuming the plant has a beneficial effect on serum glucose and insulin levels and its efficacy in weight management has been demonstrated through in vitro experiments and clinical populations [4,5]. The bioactive compound content varies considerably due to several factors, including genetic profile, phenological stage, and environmental stress exposure [6,7,8]. However, the molecular mechanisms underlying the metabolic regulation of *Opuntia* spp. have not been elucidated.

Proteomic analysis is an increasingly common approach used to capture physiological changes because this method explores the dynamic nature of proteins under various conditions. Proteomic analysis using two-dimensional differential image gel electrophoresis (2D-DIGE) has contributed to the identification of stress-responsive genes in various crop species [9,10]. More recently, proteomic analyses have also been performed in gel-free conditions to compensate for the limitations of 2D-DIGE, such as poor reproducibility and low sensitivity to low copy proteins [11]. In addition, the gel-free methodology can speed up sample preparation, enabling large-scale comparisons across different developmental stages or environmental conditions [11]. Nevertheless, to the best of our knowledge, gel-free proteomic analysis has not been widely applied to succulent plants. Only one report has analyzed the chlorophyll proteome of agave after organelle isolation [12]. It is difficult to prepare succulent plant samples for gel-free proteomic analysis, which is a major limiting factor for the application of this method.

Complicated protein extraction methods have been employed to avoid interference from the high levels of complex polysaccharides in succulents. The time-consuming and labor-intensive method to prepare *Aloe vera* involves large-scale extraction, repeated ultrafiltration, and vacuum evaporation [13]. Another method for *Agave* spp. extraction entails an intricate filtration step that makes use of natural plant fibers as a filter [14]. The proteome of *Opuntia* spp. was successfully analyzed; however, the method was not gel-free, containing gel electrophoresis as the last step [15]. The development of more rapid and simple methods for succulent sample preparation will facilitate experimental processes and research.

In this study, we developed a phenol extraction method for preparing cladodes of *Opuntia ficus-indica* (L.) Miller, the most domesticated and economically important species in the genus, for proteomic analysis. The aim of this study was to optimize protein extraction to facilitate gel-free proteomic analysis of *Opuntia* spp. In addition, we investigated whether this method is sensitive enough to capture physiological changes under environmental stresses by applying our novel gel-free proteomic analysis method to monitor the responses of *O. ficus-indica* to salt stress.

## 2. Results and Discussion

### 2.1. The Optimization of the Protein Extraction Method for Gel-Free Proteomic Analysis

**Figure 1:**
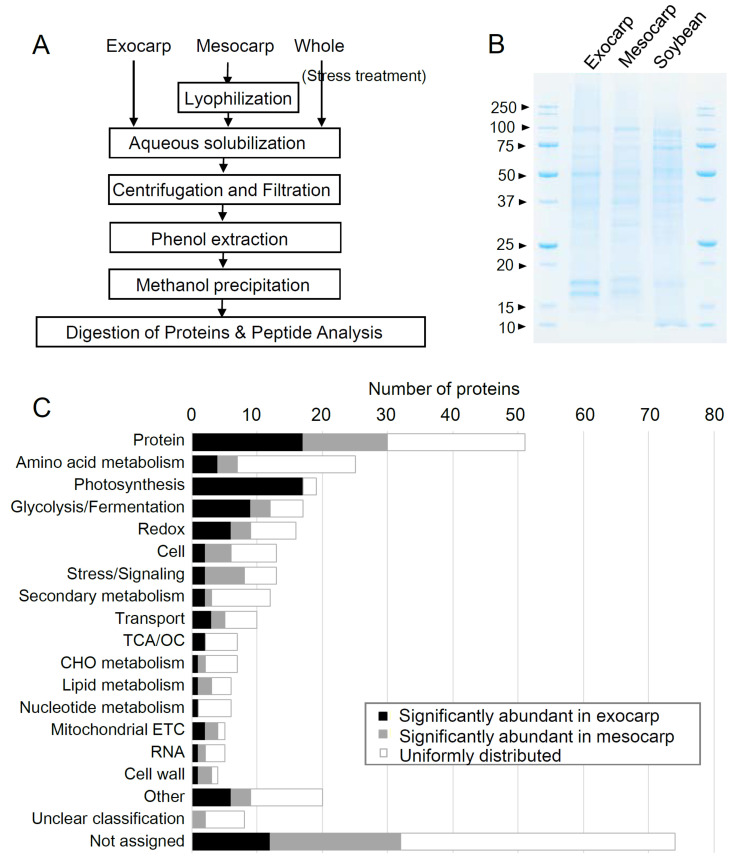
(**A**) Our optimized protein extraction method enables LC–MS/MS analysis of *Opuntia* proteins without electrophoretic separation. (**B**) Comparison of the optimized protein extraction method for *O. ficus-indica* and the TCA/acetone method applied to soybean. Proteins (5 μg for each sample) were visualized with Coomassie Brilliant Blue stain. (**C**) The number of detected proteins belonging to each functional category is shown. Differentially abundant proteins between tissues are grouped. Abbreviations: TCA/OC, tricarboxylic acid/organic acid transformations; CHO metabolism, carbohydrate metabolism; mitochondrial ETC, mitochondrial electron transport chain. Other includes development, C1 metabolism, hormone metabolism, biodegradation of xenobiotics, DNA, N-metabolism, oxidative pentose phosphate pathway, and miscellaneous function.

The succulent cladodes of *O. ficus-indica* (Supplementary Materials Table S1) contain high levels of polysaccharides that constrain protein purification from this plant. Protein precipitation using TCA/acetone is a common sample preparation strategy for the gel-free proteomic analysis of major crops, such as soybean [16]. In this study, TCA/acetone precipitation produced a sticky protein pellet, but the remaining polysaccharides presented an obstacle to achieving protein solubilization by the lysis buffer. To provide high-quality protein samples, we removed the polysaccharides using a combination of aqueous solubilization, filtration with 0.45-μm filter, and purification with alkaline phenol (Figure 1A).

After the phenol extraction, a white pellet was obtained. The reproducibility of extraction was evaluated using four technical replicates prepared from one cladode, showing similar SDS-PADE patterns to each other (Supplementary Materials Figure S1). This modified extraction method produced protein patterns that were highly reproducible with defined bands in the 17–100 kDa range from both the exocarp and mesocarp samples (Figure 1B). No low-molecular-weight smear was observed, suggesting that protein degradation did not occur. The band sharpness was similar to that obtained from soybean samples prepared by TCA/acetone precipitation. Thus, this method is an efficient protocol for extracting proteins from *Opuntia* tissues; the resulting samples were suitable for direct LC–MS/MS analysis without further purification (Figure 1A).

LC–MS/MS analysis was used to produce a catalog of proteins extracted from the *Opuntia* exocarp and the mesocarp samples. Using the SwissProt Viridiplantae database, 319 proteins were identified with ≥2 unique peptides (Supplementary Materials Table S2). 

The number of identified proteins was smaller than those in a previous gel-based report, which ranged from 590–1506 among different *Opuntia* species [15]. Filter-based purification might have reduced the protein species in the samples. Yet, major proteispecies identified in the previous study were also detected in this study (45%, 57/127 proteins), suggesting that this rapid method done without time-consuming in-gel digestion is useful for analyzing *Opuntia* proteome. Functional classification of these proteins revealed that protein homeostasis was the most represented function followed by amino acid metabolism (Figure 1C). Regarding the nutritional value of *O. ficus-indica*, some enzymes involved in secondary metabolism were detected (Supplementary Materials Table S2). Enzymes related to the biosynthesis of health-promoting compounds are difficult to detect because they are often present in relatively low levels, especially enzymes lying downstream of sequential reactions [17]. Because *O. ficus-indica* is characterized by flavonoid-like compounds such as nicotiflorin and narcissin, the removal of highly abundant proteins is required for further evaluation of the functional food resource potential of this plant [6]. Comparative analysis of diverse *Opuntia* spp. will facilitate the identification of characteristic metabolic pathways, accelerating the development of new value-adding varieties.

**Figure 2:**
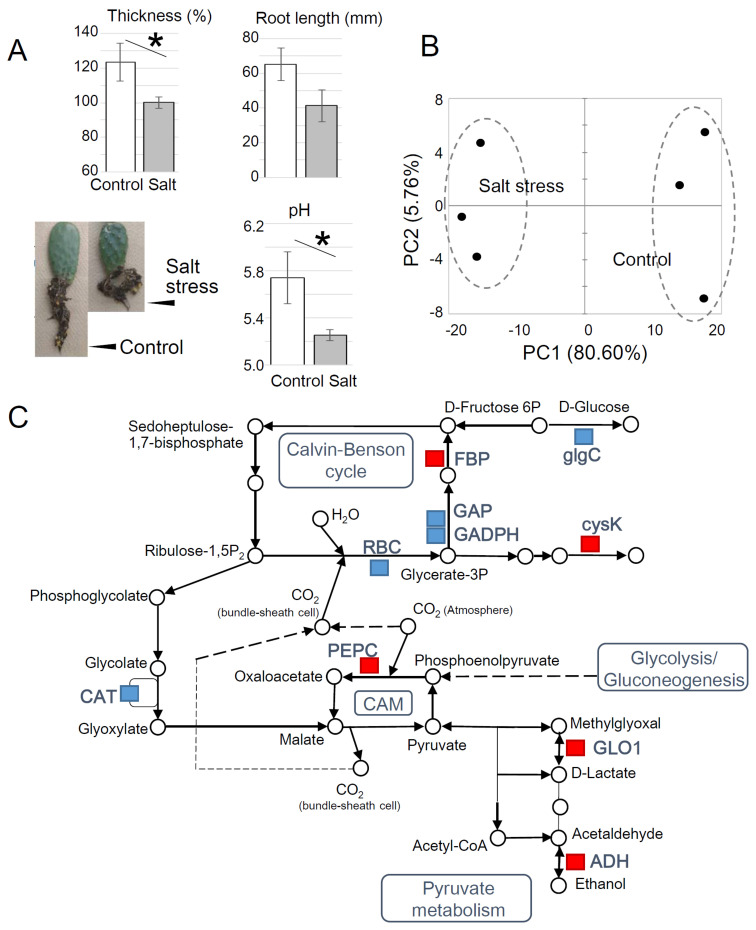
(**A**) Morphological changes of young *O. ficus-indica* plants grown from cladode cuttings after 11 days of salt stress treatment. Cladode thickness is expressed as the ratio to the value at the beginning of stress treatment. The pH of the hot water cladode tissue extracts was measured using a glass electrode pH meter. Each bar represents the mean ± S.D. (*n* = 5 independent experiments) (* *p* < 0.05). (**B**) Principal component analysis of the change in plant protein profiles in response to salt stress. Each sample had three biological replicates. (**C**) Pathway mapping of the affected proteins. Proteins that increased or decreased in abundance with salt stress are shown in red and blue boxes, respectively.

Comparing the protein abundance between tissues (*p* < 0.05, fold change cutoff at 1.5 (>|0.5849|)) revealed that the proteins accumulated in the exocarp were mainly related to energy metabolism, namely photosynthesis and glycolysis/fermentation, in addition to protein homeostasis (Figure 2C). In the mesocarp, proteins related to stress/signaling and cellular processes were dominant (Figure 2C). The presence of calcium-related proteins, such as calmodulin and calreticulin, was also noteworthy because the cladodes contain substantial amounts of calcium that can bind to chaperones in a stress response (Supplementary Materials Table S2) [18,19]. These results demonstrate that our optimized protein extraction method permitted high sensitivity for detecting proteins in both the exocarp and the mesocarp.

### 2.2. Opuntia Ficus-Indica Salt Stress Response

To determine whether this method is sensitive enough to capture physiological adaptations to environmental stress, we monitored the morphological changes in young plants grown from cladode cuttings. A 19% decline in cladode thickness relative to the control was observed after 11 days of salt stress (Figure 2A). The pH of hot water cladode extracts was measured [20] and the resulting differences suggested that malate metabolism was altered in response to salt stress (Figure 2A). To characterize the effects of salt stress on the physiological status, the cladodes were collected in the morning, and proteins were subjected to LC–MS/MS analysis. In total, 39 proteins were differentially accumulated (*p* < 0.05, fold change cutoff at 1.5 (>|0,5849|)) (Supplementary Materials Table S3). Principle component analysis (PCA) was used to assess the effects of salt stress (Figure 2B). In the scores along the direction of the first two principal component axes (PC1 and PC2), samples from plants subjected to salt stress were classified as different from control with a cumulative contribution of 86.4%, confirming that the stress intensity and the treatment period altered the physiological status of *Opuntia* (Figure 2B).

The differentially accumulated proteins were mapped on Kyoto Encyclopedia of Genes and Genomes (KEGG) pathway maps to visualize the influence of salt stress. Changes in sugar metabolism were shown by an increased protein abundance of fructose-1,6-bisphosphatase (FBP), phosphoenolpyruvate carboxylase (PEPC), alcohol dehydrogenase (ADH), and two other enzymes (Figure 2C). Conversely, the abundance of enzymes involved in the Calvin–Benson cycle, such as glyceraldehyde-3-phosphate dehydrogenase (GAP), glyceraldehyde-3-phosphate dehydrogenase (GAPDH), and ribulose-bisphosphate carboxylase (RBC), decreased in response to salt stress (Figure 2C). Among the mapped enzymes, the width of the increase or decrease in protein abundance was highest in PEPC and RBC, respectively (Supplementary Materials Table S3).

CAM photosynthesis is characterized by temporal compartmentation of carboxylation, involving primary CO_2_ fixation by PEPC in the dark and secondary CO_2_ assimilation by RBC under light conditions [21]. The differential analysis results demonstrated that salt stress impacts CAM by enhancing primary CO_2_ fixation. Circadian rhythmicity of the CAM pathway metabolite levels has been reported [22,23]; however, a previous study showed that PEPC transcript accumulation patterns in *O. ficus-indica* were arrhythmic [23]. In this study, a change in PEPC protein abundance was captured, illustrating the importance of CAM machinery regulation at the protein level (Supplementary Materials Table S3; Figure 2C).

## 3. Materials and Methods

### 3.1. Plant Materials

The donor *O. ficus-indica* plants, which have been maintained at the University of Tsukuba since 1990 for experimental purposes, were grown under natural environmental conditions in Tsukuba, Japan. Cladode tissue was excised from plants in June 2019 and prepared as specimens to optimize our protein extraction method for gel-free proteomic analysis. The exocarp and mesocarp samples were excised from three different cladodes from three different plants as biological replicates. The elemental composition of the cladode tissue was measured using a UNICUBE elemental analyzer (Elementar, Langenselbold, Hessen, Germany). For the salt stress response analysis, young cladode cuttings were collected from plants from May–June 2020, transported to the laboratory, and planted in slit pots (100 mL) with a mixture of pumice, Akadama (red granular) soil, and Kanuma trass (1:1:1). Pots were maintained in a growth chamber at 23 °C under a 16 h light (300 μmol m^−2^ s^−1^ PPFD) and 8 h dark cycle. Plants were watered once a week after rooting. Starting from the fourth week after planting, salt stress was induced as previously described by placing the pots on a plastic container filled with water containing 250 mM NaCl until the water rose from the bottom slits to the surface of the pot soil once every three days [24]. Samples subjected to salt stress were prepared from three different plantlets as biological replicates. The pH of the hot water cladode extracts was measured as described previously to estimate the malate content [20]. Briefly, cladode tissues (1 g) were ground in liquid nitrogen, put in test tubes with distilled water, and heated by microwave oven for 2 min. The extracts were brought up to 20 mL with distilled water. After filtration with a cheesecloth, the pH of the extracts was directly measured with a glass electrode pH meter.

### 3.2. Protein Extraction, Enrichment, and Digestion for Mass Spectrometry Analysis

In this work, we present an optimized protein extraction method for gel-free proteomic analysis of *O. ficus-indica*. Briefly, cladode tissue samples (1 g) were ground and transferred to a 15 mL solution of 100 mM Tris-HCl (pH 8.0), 1.5 mM potassium chloride, 10 mM dithiothreitol, 1 mM phenylmethylsulfonyl fluoride, and 0.1% SDS. The resulting suspension was sonicated for 20 min, followed by centrifugation at 4 °C for 20 min at 13,000× *g*, after which the supernatant was carefully collected to exclude the gelatinous polysaccharides. Collecting only the thin, clear supernatant is of vital importance to the success of this extraction process. Lyophilization of the tissue sample reduced the viscosity of gelatinous substances, enhancing our ability to separate the supernatant, especially when mesocarp tissue was used. After the supernatant was passed through a 0.45-μm filter (Millipore, Billerica, MA, USA), an equal volume of Tris-HCl-saturated phenol (pH 8.0) was added and mixed well by vigorous vortexing. Next, the mixture was centrifuged at 4 °C for 30 min at 3500× *g* and the top phenol phase was collected. The proteins were precipitated by adding three volumes of cold methanol containing 0.1  M ammonium acetate at −20 °C for 2  h. The precipitated proteins were recovered by centrifugation at 4 °C for 20 min at 13,000× *g* and then washed three times with cold methanol containing 0.1  M ammonium acetate. The protein pellet was air-dried and solubilized in a lysis buffer containing 7 M urea, 2 M thiourea, 5% CHAPS, and 2 mM tributylphosphine. The protein concentration was determined using a Bradford assay with bovine serum albumin as the standard. Next, the proteins (100 μg) were enriched with methanol and chloroform to remove any remaining detergent from the sample solutions. The reduction and alkylation of proteins were performed as described previously [16]. The supernatant was collected and analyzed using nanoscale liquid chromatography with tandem mass spectrometry (LC–MS/MS), as described in Section 3.3. Three independent experiments were performed as biological replicates.

### 3.3. Nanoliquid Chromatography–Tandem Mass Spectrometry Analysis

The peptides were loaded onto the LC system (EASY-nLC 1000; Thermo Fisher Scientific, San Jose, CA, USA) equipped with a trap column (Acclaim PepMap 100 C18 LC column, 3 μm, 75 μm ID × 20 mm; Thermo Fisher Scientific), equilibrated with 0.1% formic acid, and eluted with a linear acetonitrile gradient (0–35%) in 0.1% formic acid at a flow rate of 300 nL min^−1^. The eluted peptides were loaded and separated on an EASY-Spray C18 LC column (3 μm, 75 μm ID × 150 mm; Thermo Fisher Scientific, San Jose, CA, USA) with a spray voltage of 2 kV (ion transfer tube temperature: 275 °C). The peptide ions were detected using MS (Orbitrap Fusion ETD MS; Thermo Fisher Scientific, San Jose, CA, USA) in the data-dependent acquisition mode using the installed Xcalibur software (version 4.0; Thermo Fisher Scientific, San Jose, CA, USA). Full-scan mass spectra were acquired using a Fourier-transform (FT) MS over 375–1500 m/z with a resolution of 120,000. The most intense precursor ions were selected for collision-induced dissociation (CID) at a normalized collision energy of 35%. Dynamic exclusion was employed within 60 s to prevent repetitive selection of peptides.

### 3.4. Analysis of the Differential Abundance of Proteins Acquired Using Mass Spectrometry

Proteins in the samples were identified from the SwissProt Viridiplantae database (25 October 2017) using the MASCOT (version 2.6.1, Matrix Science, London, UK) and Sequest HT search engine. The acquired raw data files were processed using Proteome Discoverer software 2.2 (version 2.2.0.388; Thermo Fisher Scientific, San Jose, CA, USA) using precursor ions quantifiler nodes. Target-decoy database searches used for the calculation of false discovery rate (FDR) and for peptide identification FDR was set at 1%. PERSEUS software (version 1.6.10.43) [25] was used for differential analysis of the relative abundance of peptides based on extracted ion chromatogram (XIC). Abundance of proteins was estimated based on all peptides (FDR < 1%) contributing to an individual protein [25]. Proteins with ≥2 unique peptides were selected as identified. Duplicated protein identifications resulting from orthologous proteins in the database were removed manually.

### 3.5. Bioinformatic and Statistical Analyses

Proteins were categorized based on function using MapMan bin codes [26]. To determine which biological processes are affected by salt stress, the differently abundant proteins were mapped using the Kyoto Encyclopedia of Genes and Genomes (KEGG) pathways [27]. Statistical significance was evaluated by one-way ANOVA using SPSS statistical software (version 23.0; IBM, Armonk, NY, USA). *p*-values less than 0.05 were considered statistically significant. Principle component analysis (PCA) was performed using XLSTAT software, comparing the mean logarithmically transformed protein abundance ratios with the non-treated control.

## 4. Conclusions

In the current study, a protein extraction method that accommodates gel-free quantitative workflow was established for *O. ficus-indica*. Successful gel-free proteomic analysis was performed for the first time using this plant. Cladode proteins from the exocarp and the mesocarp were analyzed separately and in combination. Functional differentiation between tissues was recognized, and the environmental control of CAM in response to salt stress was demonstrated in young plants using pot experiments. The method established in this study will facilitate large-scale gel-free analyses of succulent proteomes. The impact of environmental factors on the quality of *O. ficus-indica* will be investigated in our future work using proteome analysis to determine physiological responses.

## Data Availability

The mass spectrometry data have been deposited to the ProteomeXchange Consortium with the dataset identifier PXD022276.

## Supplementary Materials

The following are available online at https://www.mdpi.com/2223-7747/10/1/115/s1, Table S1: Elemental composition of cladode tissues, Table S2: List of cladode proteins, Table S3: List of affected proteins by salt stress. Figure S1: SDS-PAGE gel of exocarp proteins.
