# Supplementary material for: An Optimized Protein Extraction Method for Gel-Free Proteomic Analysis of Opuntia Ficus-Indica
Source: Plants (Basel). 2021 Jan 8;10(1):115. doi: 10.3390/plants10010115 (PMC7827026; doi:10.3390/plants10010115)
Supplement: Supplementary file 1 [file plants-10-00115-s001.pdf]

Supplementary: **"An optimized protein extraction method for gel-free proteomic analysis of Opuntia ficus-indica "**

Supplementary Table 1. Elemental composition of cladode tissues.

Tissue	CHNS analysis				Yield [2]
	C [1,2]	H [1,2]	N [1,2]	S [1,2]	
Exocarp	34.0 ± 2.94*	4.7 ± 0.55	1.12 ± 0.11*	0.5 ± 0.12	9.8 ± 0.01*
Mesocarp	27.2 ± 1.88*	3.8 ± 0.33	0.8 ± 0.15*	0.6 ± 0.11	2.4 ± 0.00*

1 Values are the mean of triplicates expressed on dry weight basis ± standard deviation.

2 Expressed as %.

*p<0.05 in the same column.

Supplementary Table 2. List of cladode proteins

	Accession[a]	EC Number	Description	Cover (%)b	M.P.[c]	Exocarp		Mesocarp		*p*-value	Difference[e]	Disrtibution[f]	Function[g]
						Abd[d]	S.D.	Abd[d]	S.D.				
1	A0A0J8B5Q5	EC 1.4.4.2	glycine cleavage system P protein [OS=Beta vulgaris subsp. vulgaris]	11	2	1.7	0.33	-4.4	1.20	0.001	6.08	E	Amino acid metabolism
2	A0A5P8N5L8	-	AtpB (Fragment) OS=Hymenophyllum polyanthos OX=160852 PE=4 SV=1	57	2	1.2	0.33	-3.9	0.63	0.000	5.07	E	Mitochondrial electron transport / ATP synthesis
3	A0A1S3UU57	-	PGR5-like protein 1A, chloroplastic [OS=Vigna radiata var. radiata]	7	2	1.2	0.24	-3.2	0.96	0.002	4.43	E	Photosynthesis
4	A0A2P5B3A0	EC 5.2.1.8	Peptidyl-prolyl cis-trans isomerase OS=Parasponia andersonii OX=3476 GN=PanWU01x14_275960 PE=3 SV=1	10	2	0.0	0.53	-4.2	1.25	0.006	4.16	E	Cell
5	Q9FUJ4	EC 4.1.1.39	Ribulose bisphosphate carboxylase small chain [OS=Glycine max]	18	3	1.0	0.17	-2.8	0.00	0.000	3.85	E	Photosynthesis
6	A0A0A7M862	EC 3.6.5.3	elongation factor 1-alpha [OS=Pinus massoniana]	25	2	1.4	0.28	-2.2	0.98	0.003	3.67	E	Protein
7	J7I3K0	EC:1.4.1.14	glutamate synthase [OS=Beta vulgaris]	10	2	1.8	0.31	-1.8	0.07	0.000	3.62	E	N-metabolism
8	A0A5A7PJT9	EC 2.1.2.1	Serine hydroxymethyltransferase OS=Striga asiatica OX=4170 GN=STAS_09221 PE=3 SV=1	14	2	1.9	0.18	-1.5	0.44	0.000	3.36	E	Photosynthesis
9	A0A1D1Z7U1	-	inner envelope membrane protein, chloroplastic [OS=Anthurium amnicola]	10	2	0.6	0.54	-2.7	0.60	0.002	3.29	E	Protein
10	A0A1Q3BTG8	EC 1.1.1.37	malate dehydrogenase [OS=Cephalotus follicularis]	31	3	0.9	0.30	-2.4	1.97	0.047	3.27	E	Fermentation
11	A0A165XZJ9	EC 1.2.1.-	glyceraldehyde-3-phosphate dehydrogenase [OS=Daucus carota subsp. sativus]	27	4	5.0	0.47	1.8	0.19	0.000	3.26	E	Photosynthesis
12	A0A0K9QBI6	EC 2.1.2.10	aminomethyltransferase [OS=Spinacia oleracea]	18	2	2.2	0.01	-1.0	0.02	0.000	3.23	E	Amino acid metabolism

13	A0A2C9W771	EC:3.1.4.46	Glycerophosphodiester phosphodiesterase OS=Manihot esculenta OX=3983 GN=MANES_03G134600 PE=4 SV=1	3	2	2.8	0.36	-0.4	0.82	0.004	3.17	E	Lipid metabolism
14	Q43157	EC 5.1.3.1	Ribulose-phosphate 3-epimerase, chloroplastic [OS=Spinacia oleracea]	13	2	3.6	0.32	0.6	0.36	0.000	3.06	E	Photosynthesis
15	A0A498HI53	-	Aldo_ket_red domain-containing protein OS=Malus domestica OX=3750 GN=DVH24_018553 PE=4 SV=1	6	2	1.5	0.58	-1.5	0.85	0.007	3.05	E	Redox
16	A0A1S4DDU7	-	nucleosome assembly protein 11-like [OS=Nicotiana tabacum]	10	3	3.6	0.71	0.6	0.78	0.008	2.96	E	DNA
17	Q36795	EC 7.4.2.4	Protein translocase subunit secA, chloroplastic [OS=Spinacia oleracea]	8	2	1.0	0.25	-2.0	0.30	0.000	2.91	E	Protein
18	A0A1S3VRE4	EC:2.6.1.4; 2.6.1.2; 2.6.1.44	glutamate--glyoxylate aminotransferase 2 [OS=Vigna radiata var. radiata]	20	2	1.3	0.29	-1.6	0.48	0.001	2.89	E	Amino acid metabolism
19	A0A0V0ID49	-	Putative phenylalanine--tRNA ligase, chloroplastic/mitochondrial-like [OS=Solanum chacoense]	5	2	-0.7	0.43	-3.5	1.05	0.012	2.83	E	Protein
20	K3YIU7	EC 1.15.1.1	Superoxide dismutase [Cu-Zn] [OS=Setaria italica]	9	2	0.7	0.48	-2.1	0.17	0.001	2.80	E	Redox
21	H2BBA9	EC 7.1.2.2	ATP synthase subunit alpha, chloroplastic [OS=Opuntia decumbens]	37	6	8.2	0.29	5.5	0.17	0.000	2.74	E	Photosynthesis
22	A0A1R3KJF8	-	Chaperonin Cpn60 [OS=Corchorus olitorius]	32	3	1.7	0.64	-0.8	1.10	0.028	2.48	E	Protein
23	A0A061GTT9	-	Alanine:glyoxylate aminotransferase isoform 1 [OS=Theobroma cacao]	30	2	4.3	0.25	1.9	0.30	0.000	2.46	E	Photosynthesis
24	B9SW87	-	mitochondrial carrier protein, putative [OS=Ricinus communis]	13	2	-2.0	0.86	-4.4	0.92	0.032	2.34	E	Not assigned
25	A0A1U8EH95	-	ribulose bisphosphate carboxylase/oxygenase activase 1, chloroplastic-like [OS=Capsicum annuum]	28	2	4.4	0.08	2.1	0.30	0.000	2.32	E	Photosynthesis
26	H6TH77	C:1.10.3.9	Photosystem II D2 protein [OS=Triglochin maritima]	23	2	6.3	0.23	3.9	0.69	0.005	2.31	E	Photosynthesis
27	A0A0K9Q369	EC:2.2.1.7	1-deoxy-D-xylulose-5-phosphate synthase, transketolase [OS=Zostera marina]	10	2	1.5	0.56	-0.8	0.66	0.011	2.27	E	Secondary metabolism

28	A0A218Y336	EC 2.7.1.11	ATP-dependent 6-phosphofructokinase [OS=Punica granatum]	11	2	2.8	0.16	0.6	0.27	0.000	2.26	E	Glycolysis
29	H6T935	-	photosystem I P700 apoprotein A1 [OS=Opuntia decumbens]	12	2	3.6	0.30	1.3	0.76	0.009	2.25	E	Photosynthesis
30	A0A1R3GLZ3	-	ATPase, AAA-type, core [OS=Corchorus capsularis]	32	3	5.0	0.48	2.8	0.51	0.005	2.22	E	Protein
31	X2CQ79	-	protein synthesis elongation factor Tu [OS=Chlorella sp. 2009100806]	10	2	2.8	0.05	0.6	0.07	0.000	2.20	E	Protein
32	W9S244	EC:1.23.5.1	Violaxanthin de-epoxidase [OS=Morus notabilis]	5	2	-0.4	0.46	-2.6	0.73	0.012	2.18	E	Secondary metabolism
33	A0A5D2E6D3	-	FAD-binding FR-type domain-containing protein OS=Gossypium darwinii OX=34276 GN=ES288_A12G065500v1 PE=4 SV=1	4	2	2.9	0.29	0.7	0.16	0.000	2.14	E	Miscellaneous
34	A0A103YEZ0	-	Electron transport accessory protein-like domain-containing protein [OS=Cynara cardunculus var. scolymus]	24	3	4.4	1.00	2.3	0.02	0.022	2.10	E	Not assigned
35	A0A4P1RSA7	-	Epimerase domain-containing protein OS=Lupinus angustifolius OX=3871 GN=TanjilG_22577 PE=4 SV=1	15	2	-1.8	0.71	-3.9	0.24	0.008	2.10	E	Not assigned
36	Q95633	EC 4.1.1.39	Ribulose bisphosphate carboxylase large chain (Fragment) OS=Brucea mollis OX=43723 GN=rbcL PE=3 SV=3	24	2	-1.8	0.66	-3.8	0.52	0.014	2.03	E	Photosynthesis
37	A0A1S4A548	-	stromal 70 kDa heat shock-related protein, chloroplastic-like [OS=Nicotiana tabacum]	26	2	2.2	0.70	0.2	0.43	0.013	2.00	E	Stress
38	A0A6A1WJU5	-	Thylakoid lumenal 19 kDa protein, chloroplastic OS=Morella rubra OX=262757 GN=CJ030_MR1G017681 PE=4 SV=1	10	2	1.4	0.81	-0.6	0.39	0.019	1.99	E	Not assigned
39	A0A124SGQ3	EC:2.1.1.-	fibrillarin [OS=Cynara cardunculus var. scolymus]	12	2	-1.0	0.37	-2.9	0.54	0.007	1.92	E	Protein
40	A0A072TW15	EC 6.3.5.2	GMP synthase [glutamine-hydrolyzing] protein [OS=Medicago truncatula]	6	2	-1.8	0.35	-3.7	0.56	0.008	1.89	E	Nucleotide metabolism

41	H6T8S0	-	Cytochrome f [OS=Opuntia decumbens]	32	5	4.3	0.23	2.4	0.23	0.001	1.86	E	Redox
42	A0A151TMH0	EC 1.13.11.-	Lipoxygenase [OS=Cajanus cajan]	5	2	2.2	0.17	0.4	0.43	0.002	1.86	E	Hormone metabolism
43	D2KLH0	EC:1.11.1.11	thylakoid ascorbate peroxidase [OS=Suaeda salsa]	16	5	1.9	0.24	0.0	0.32	0.001	1.84	E	Redox
44	A0A151S6X6	-	40S ribosomal protein S18 [OS=Cajanus cajan]	30	2	-0.4	0.42	-2.2	0.38	0.005	1.83	E	Protein
45	J9WR78	EC 2.4.1.13	sucrose synthase [OS=Amaranthus hypochondriacus]	5	2	-0.3	0.60	-2.1	0.16	0.007	1.81	E	CHO metabolism
46	V5PZT7	-	mitochondrial dicarboxylate transporter [OS=Suaeda glauca]	19	2	-1.1	0.57	-2.9	0.13	0.006	1.80	E	Transport
47	W9RGS5	EC:2.2.1.1	Transketolase [OS=Morus notabilis]	28	2	3.6	0.81	1.8	0.12	0.019	1.79	E	Photosynthesis
48	A0A1U8LXQ6	-	Photosystem II CP47 reaction center protein [OS=Gossypium hirsutum]	17	3	8.0	0.15	6.2	0.15	0.000	1.75	E	Photosynthesis
49	A0A444YG83	EC:3.2.1.1	1,4-alpha-D-glucan glucanohydrolase OS=Arachis hypogaea OX=3818 GN=Ahy_B06g079784 PE=3 SV=1	8	2	3.6	0.26	1.8	0.10	0.000	1.73	E	Not assigned
50	A0A059D1G7	EC 6.3.1.2	Glutamine synthetase [OS=Eucalyptus grandis]	20	2	2.9	0.53	1.2	0.87	0.042	1.73	E	N-metabolism
51	A9TKZ5	-	ATP-binding cassette transporter, subfamily G, member 2, group WBC protein PpABCG2 [OS=Physcomitrella patens]	5	2	0.1	0.18	-1.6	0.35	0.002	1.67	E	Transport
52	P12354	-	Photosystem I reaction center subunit IV, chloroplastic [OS=Spinacia oleracea]	18	2	2.6	0.28	0.9	0.09	0.001	1.67	E	Photosynthesis
53	A0A6A1UJU3	EC:4.2.1.1	Carbonic anhydrase OS=Morella rubra OX=262757 GN=CJ030_MR0G006360 PE=3 SV=1	21	2	6.8	0.26	5.1	0.21	0.001	1.65	E	TCA / org transformation
54	A0A0V0I0B9	EC:5.3.1.1	Putative triosephosphate isomerase, chloroplastic-like [OS=Solanum chacoense]	16	2	3.9	0.44	2.3	0.24	0.005	1.60	E	Glycolysis
55	A0A1E5VMJ7	-	Chaperone protein ClpC2, chloroplastic [OS=Dichanthelium oligosanthes]	25	2	4.5	0.33	2.9	0.25	0.003	1.58	E	Protein
56	F8U877	EC 2.7.7.27	glucose-1-phosphate adenylyltransferase [OS=Amorphophallus konjac]	8	3	2.5	0.16	1.0	0.60	0.012	1.58	E	Cell wall

57	A0A1U7ZUH4	-	UPF0603 protein At1g54780, chloroplastic-like [OS=Nelumbo nucifera]	7	2	2.1	0.60	0.6	0.05	0.011	1.55	E	Not assigned
58	A0A0B0PKU9	EC 2.7.1.-	phosphotransferase [OS=Gossypium arboreum]	11	2	1.9	0.23	0.4	0.35	0.003	1.53	E	Not assigned
59	A0A4S4E9Z4	-	KH type-2 domain-containing protein OS=Camellia sinensis var. sinensis OX=542762 GN=TEA_001017 PE=3 SV=1	22	2	1.0	0.03	-0.5	0.34	0.002	1.49	E	Not assigned
60	A0A5N6N5U7	-	Histone H4 OS=Mikania micrantha OX=192012 GN=E3N88_25218 PE=3 SV=1	5	7	6.2	0.76	4.7	0.22	0.032	1.48	E	RNA
61	A0A2I0W0U5	-	RuBisCO large subunit-binding protein subunit beta, chloroplastic OS=Dendrobium catenatum OX=906689 GN=MA16_Dca002552 PE=3 SV=1	33	5	5.7	0.65	4.2	0.18	0.020	1.47	E	Photosynthesis
62	A0A1R3J767	EC:1.11.1.9	Glutathione peroxidase [OS=Corchorus olitorius]	11	2	2.1	0.64	0.6	0.25	0.022	1.46	E	Redox
63	A0A061G4U7	EC:2.7.11.1	Eukaryotic translation initiation factor 2 subunit 1 [OS=Theobroma cacao]	12	3	0.9	0.18	-0.6	0.29	0.002	1.42	E	Protein
64	A0A2I4GNB3	-	K(+) efflux antiporter 2, chloroplastic-like OS=Juglans regia OX=51240 GN=LOC109009387 PE=4 SV=1	4	2	-0.2	0.14	-1.6	0.03	0.000	1.39	E	Not assigned
65	A0A2U1N5P2	EC 1.10.3.9	Multifunctional fusion protein OS=Artemisia annua OX=35608 GN=psbD PE=3 SV=1	21	2	8.3	0.49	7.0	0.07	0.010	1.32	E	Not assigned
66	G4XKY2	-	Vacuolar proton pump ATPase subunit H [OS=Suaeda corniculata]	14	2	4.3	0.37	3.0	0.11	0.004	1.30	E	Transport
67	A0A151TMX4	EC 3.4.21.92	ATP-dependent Clp protease proteolytic subunit [OS=Cajanus cajan]	11	2	3.5	0.17	2.2	0.32	0.004	1.22	E	Protein
68	A0A2R6QCJ5	EC:5.4.2.12	Phosphoglycerate mutase (2,3-diphosphoglycerate-independent) OS=Actinidia chinensis var. chinensis OX=1590841 GN=CEY00_Acc19094 PE=3 SV=1	21	2	5.6	0.32	4.5	0.41	0.017	1.18	E	Glycolysis
69	A0A5J5B9C0	EC:5.4.2.2	Phosphoglucomutase (alpha-D-glucose-1,6-bisphosphate-dependent) OS=Nyssa sinensis OX=561372 GN=F0562_026547 PE=3 SV=1	21	2	1.8	0.09	0.7	0.41	0.010	1.12	E	Glycolysis
70	A0A061GTA5	-	Myosin-related isoform 1 [OS=Theobroma cacao]	2	2	2.2	0.09	1.1	0.14	0.000	1.10	E	Cell

71	M8AT63	-	60S ribosomal protein L10-2 [OS=Triticum urartu]	5	2	3.2	0.46	2.1	0.44	0.041	1.10	E	Protein
72	A0A1U8EUA7	EC 1.2.4.1	Pyruvate dehydrogenase E1 component subunit alpha [OS=Capsicum annuum]	14	2	0.8	0.38	-0.2	0.19	0.015	1.00	E	TCA / org transformation
73	A0A5J4ZLP8	-	Ubiquitin OS=Nyssa sinensis OX=561372 GN=F0562_016032 PE=3 SV=1	23	4	4.5	0.49	3.5	0.18	0.030	0.99	E	Protein
74	A0A200PVX1	-	ribosomal protein L14 [OS=Macleaya cordata]	18	2	2.6	0.12	1.6	0.16	0.001	0.98	E	Protein
75	A0A200RA25	-	Heat shock chaperonin-binding [OS=Macleaya cordata]	12	4	2.0	0.15	1.1	0.18	0.002	0.96	E	Stress
76	A0A1R3H0S9	-	ribosomal protein S4E [OS=Corchorus capsularis]	6	5	3.7	0.01	2.8	0.14	0.000	0.93	E	Protein
77	Q42649	EC 3.1.3.11	Fructose-1,6-bisphosphatase, cytosolic [OS=Beta vulgaris]	20	3	3.5	0.37	2.6	0.20	0.022	0.89	E	Photosynthesis
78	A0A087GZ87	EC 2.5.1.47	cysteine synthase [OS=Arabis alpina]	15	2	1.9	0.21	1.0	0.19	0.005	0.89	E	Amino acid metabolism
79	A0A1S3BP61	EC:6.2.1.1	acetyl-coenzyme A synthetase, chloroplastic/glyoxysomal isoform X1 [OS=Cucumis melo]	4	2	0.7	0.21	-0.1	0.21	0.009	0.80	E	Glycolysis
80	A0A0K9S0R0	EC 2.7.2.3	Phosphoglycerate kinase [OS=Spinacia oleracea]	26	2	5.6	0.07	4.8	0.10	0.000	0.78	E	Photosynthesis
81	A0A1S3AUC7	E1.6.5.4	Monodehydroascorbate reductase 5, mitochondrial [OS=Cucumis melo]	6	2	1.5	0.25	0.7	0.06	0.007	0.75	E	Redox
82	B6SIG5	EC:5.3.1.1]	triosephosphate isomerase, cytosolic [OS=Zea mays]	27	2	5.6	0.13	4.9	0.19	0.005	0.74	E	Glycolysis
83	A0A1Q3BYB2	-	Ribosomal_S4 domain-containing protein/rve domain-containing protein/S4 domain-containing protein/TB2_DP1_HVA22 domain-containing protein/PMEI domain-containing protein [OS=Cephalotus follicularis]	3	2	2.4	0.25	1.7	0.17	0.014	0.74	E	Not assigned
84	B9RJU9	EC 5.3.1.9	glucose-6-phosphate isomerase [OS=Ricinus communis]	13	3	1.2	0.13	0.5	0.04	0.001	0.70	E	OPP
85	A0A199UR41	EC:5.4.2.12	2,3-bisphosphoglycerate-independent phosphoglycerate mutase [OS=Ananas comosus]	10	2	3.4	0.14	2.7	0.09	0.002	0.68	E	Glycolysis
86	Q43130	EC 4.2.1.11	Enolase [OS=Mesembryanthemum crystallinum]	33	4	8.5	0.08	7.9	0.07	0.000	0.68	E	Glycolysis

87	D5I3D2	-	ATPase subunit 4 [OS=Citrullus lanatus]	26	3	2.9	0.07	2.2	0.08	0.000	0.65	E	Protein
88	A0A1Q3BQ92	-	V-type proton ATPase subunit A [OS=Cephalotus follicularis]	2	2	2.4	0.27	1.8	0.12	0.021	0.64	E	Mitochondrial electron transport / ATP synthesis
89	A0A394DFJ0	-	ATP:AMP phosphotransferase OS=Lupinus angustifolius OX=3871 GN=TanjilG_14605 PE=3 SV=1	34	2	4.4	0.28	3.8	0.12	0.027	0.61	E	Not assigned
90	A0A075IYX7	EC:4.1.1.31	Phosphoenolpyruvate carboxylase (Fragment) OS=Nopalea cochenillifera OX=338184 GN=ppc-1E1e PE=4 SV=1	41	4	-4.2	0.60	0.8	0.44	0.000	-4.99	M	Glycolysis
91	P04464	-	Calmodulin [OS=Triticum aestivum]	58	2	-1.5	0.81	3.2	0.43	0.001	-4.66	M	Signalling
92	A0A0B0Q1X9	-	Putative aquaporin PIP2-2 [OS=Gossypium arboreum]	22	2	-1.0	0.08	2.8	0.18	0.000	-3.82	M	Transport
93	A0A118K071	-	Prefoldin [OS=Cynara cardunculus var. scolymus]	14	2	-2.2	1.55	1.2	0.34	0.019	-3.46	M	Protein
94	A0A2G9GSG4	EC 3.2.1.22	Alpha-galactosidase OS=Handroanthus impetiginosus OX=429701 GN=CDL12_19235 PE=3 SV=1	8	2	-4.2	0.91	-0.8	0.17	0.003	-3.45	M	CHO metabolism
95	V7BKD4	EC 2.7.1.40	pyruvate kinase [OS=Phaseolus vulgaris]	14	2	-2.7	0.27	0.7	0.67	0.001	-3.40	M	Glycolysis
96	A0A4S4DTX0	-	EF1_GNE domain-containing protein OS=Camellia sinensis var. sinensis OX=542762 GN=TEA_029496 PE=3 SV=1	10	2	-2.5	1.37	0.7	0.08	0.015	-3.26	M	Not assigned
97	A0A1Q3C7Y8	-	Thaumatin domain-containing protein/Ufm1 domain-containing protein [OS=Cephalotus follicularis]	9	2	-2.8	1.11	0.0	0.15	0.011	-2.88	M	Stress
98	A0A1U7YRF8	-	probable galactinol--sucrose galactosyltransferase 6 isoform X1 [OS=Nelumbo nucifera]	4	2	-2.9	0.99	0.0	0.16	0.008	-2.88	M	Not assigned
99	A0A0K9QAJ7	EC 5.1.3.-	Uncharacterized protein [OS=Spinacia oleracea]	17	3	-2.1	0.19	0.7	0.04	0.000	-2.82	M	Unclear classification
100	A0A2P5BU82	EC 1.1.1.22	UDP-glucose 6-dehydrogenase OS=Trema orientale OX=63057 GN=TorRG33x02_308770 PE=3 SV=1	11	2	-4.8	0.74	-2.3	1.02	0.025	-2.55	M	Cell wall

101	A0A1U8GX81	-	Quinone oxidoreductase [OS=Capsicum annuum]	11	2	-0.9	1.20	1.5	0.75	0.042	-2.41	M	Mitochondrial electron transport / ATP synthesis
102	A0A068US01	-	small ubiquitin-related modifier [OS=Coffea canephora]	53	2	-0.8	0.32	1.6	0.29	0.001	-2.40	M	Protein
103	A0A1U8AEQ0	EC:1.1.1.1	Alcohol dehydrogenase 1-like [OS=Nelumbo nucifera]	12	2	-0.9	0.55	1.4	0.08	0.002	-2.27	M	Fermentation
104	A0A1U8GAD8	-	Probable serine/threonine-protein kinase At4g35230 [OS=Capsicum annuum]	15	3	-0.3	0.27	1.9	0.17	0.000	-2.24	M	Not assigned
105	A0A126WWJ8	-	putative LOV domain-containing protein [OS=Lophophora williamsii]	12	5	0.1	0.62	2.3	0.12	0.004	-2.17	M	Not assigned
106	A0A2P5BQB8	-	Cytochrome c oxidase, subunit Vb OS=Parasponia andersonii OX=3476 GN=PanWU01x14_220080 PE=4 SV=1	16	2	-0.2	1.02	1.7	0.39	0.036	-1.96	M	Redox
107	A0A200R7H0	-	NADPH-dependent FMN reductase [OS=Macleaya cordata]	16	3	-0.3	0.70	1.6	0.35	0.013	-1.93	M	Secondary metabolism
108	A0A1U7ZBG4	-	CSC1-like protein ERD4 [OS=Nelumbo nucifera]	3	2	0.1	1.12	2.0	0.27	0.044	-1.92	M	Not assigned
109	A0A0A8WK73	-	Aquaporin PIP1 4 [OS=Nicotiana tabacum]	8	2	1.4	0.98	3.3	0.23	0.034	-1.84	M	Transport
110	A0A4D8ZQE4	-	Clathrin heavy chain OS=Salvia splendens OX=180675 GN=CLTC PE=3 SV=1	17	2	0.0	0.36	1.9	0.13	0.001	-1.84	M	Protein
111	O82564	-	Actin 2 [OS=Anemia phyllitidis]	31	3	1.6	0.44	3.5	0.30	0.004	-1.82	M	Cell
112	F6H7L5	EC 4.4.1.5	lactoylglutathione lyase [OS=Vitis vinifera]	11	2	1.3	1.00	3.1	0.32	0.041	-1.80	M	Not assigned
113	A0A1U7ZR49	EC:6.2.1.64	NEDD8-activating enzyme E1 catalytic subunit [OS=Nelumbo nucifera]	12	3	-4.6	0.47	-2.9	0.38	0.007	-1.78	M	Protein
114	B9IQ20	EC 4.1.1.112; EC 4.1.3.17	4-hydroxy-4-methyl-2-oxoglutarate aldolase [OS=Populus trichocarpa]	18	3	0.8	0.64	2.5	0.14	0.010	-1.73	M	Biodegradation of Xenobiotics
115	W9QVF5	-	1-phosphatidylinositol phosphodiesterase [OS=Morus notabilis]	8	2	0.0	0.33	1.7	0.21	0.002	-1.66	M	Not assigned
116	A0A1Q3D7Z8	-	Calreticulin domain-containing protein [OS=Cephalotus follicularis]	17	3	-4.1	0.66	-2.5	0.69	0.041	-1.65	M	Signalling
117	A0A1D1Z5K4	EC 1.11.1.15	Peroxiredoxin-2C [OS=Anthurium amnicola]	30	2	0.9	0.74	2.5	0.32	0.025	-1.62	M	Redox

118	A0A1B1V4Y2	-	FT-1 [OS=Haloxylon ammodendron]	18	3	1.0	0.36	2.6	0.30	0.004	-1.61	M	Not assigned
119	A0A0K9S0F9	EC 2.4.1.1	alpha-1,4 glucan phosphorylase [OS=Spinacia oleracea]	16	2	2.2	0.40	3.8	0.11	0.003	-1.59	M	Not assigned
120	A0A6A2WL58	-	GTP-binding protein SAR1B OS=Hibiscus syriacus OX=106335 GN=F3Y22_tig00116958pilonHSYRG00271 PE=3 SV=1	24	2	0.5	0.05	2.1	0.37	0.002	-1.59	M	Protein
121	A0A059BK11	-	Polyadenylate-binding protein [OS=Eucalyptus grandis]	8	2	-2.8	0.41	-1.2	0.61	0.020	-1.58	M	RNA
122	A0A444YR86	EC:2.1.1.14	5-methyltetrahydropteroyltriglutamate--homocysteine S-methyltransferase OS=Arachis hypogaea OX=3818 GN=Ahy_B06g084100 PE=3 SV=1	11	2	-0.1	0.24	1.5	0.10	0.000	-1.57	M	Amino acid metabolism
123	A0A218WXL0	-	Transmembrane 9 superfamily member [OS=Punica granatum]	4	2	-1.9	0.67	-0.4	0.66	0.047	-1.54	M	Not assigned
124	A0A0K9R1J6	-	beta-adaptin-like protein [OS=Spinacia oleracea]	10	6	-0.5	0.72	1.0	0.20	0.024	-1.52	M	Cell
125	Q6PQ37	-	ubiquitin-conjugating enzyme family protein [OS=Tamarix androssowii]	34	2	-1.9	0.18	-0.4	0.53	0.010	-1.50	M	Protein
126	A0A200QHB1	EC 1.17.1.9	formate dehydrogenase, mitochondrial [OS=Macleaya cordata]	24	2	2.2	0.40	3.6	0.18	0.005	-1.44	M	C1-metabolism
127	D7SJ83	-	WD_REPEATS_REGION domain-containing protein OS=Vitis vinifera OX=29760 GN=VIT_17s0000g02670 PE=4 SV=1	8	2	-0.9	0.63	0.5	0.18	0.020	-1.43	M	Not assigned
128	A0A1U8FFB2	-	Protein arginine N-methyltransferase [OS=Capsicum annuum]	8	3	-2.4	0.46	-0.9	0.20	0.008	-1.41	M	Miscellaneous
129	K4AMY6	EC 1.5.1.20	Methylenetetrahydrofolate reductase [OS=Setaria italica]	11	2	-0.9	0.06	0.5	0.60	0.017	-1.38	M	Not assigned
130	A0A0E0AWE1	EC 4.1.1.15	glutamate decarboxylase [OS=Oryza glumipatula]	14	2	-0.1	0.60	1.2	0.06	0.020	-1.30	M	Amino acid metabolism
131	A0A0C9RGH3	-	TSA: Wollemia nobilis Ref_Wollemi_Transcript_28244_2339 transcribed RNA sequence [OS=Wollemia nobilis]	6	2	1.6	0.46	2.9	0.13	0.011	-1.25	M	Not assigned

132	A0A0J8FHR1	-	t-SNARE coiled-coil homology domain-containing protein OS=Beta vulgaris subsp. vulgaris OX=3555 GN=BVRB_3g059810 PE=4 SV=1	11	2	-2.9	0.55	-1.7	0.25	0.025	-1.21	M	Cell
133	A0A1U8JW28	EC:2.7.11.1	pto-interacting protein 1-like isoform X1 [OS=Gossypium hirsutum]	16	2	-1.5	0.13	-0.3	0.26	0.002	-1.15	M	Stress
134	A0A0B2PK57	-	GTP-binding nuclear protein [OS=Glycine soja]	38	2	4.6	0.09	5.8	0.07	0.000	-1.13	M	Not assigned
135	A0A0E0CGF3	EC 1.2.1.24	Uncharacterized protein [OS=Oryza meridionalis]	8	3	0.2	0.41	1.3	0.34	0.021	-1.13	M	Unclear classification
136	A0A0K9PKP1	-	putative ADP-ribosylation factor [OS=Zostera marina]	34	2	0.0	0.43	1.1	0.20	0.017	-1.09	M	Protein
137	A0A2P6QU17	EC 1.1.1.271	GDP-L-fucose synthase OS=Rosa chinensis OX=74649 GN=RchiOBHm_Chr4g0405311 PE=3 SV=1	12	2	-1.6	0.08	-0.5	0.33	0.005	-1.09	M	Not assigned
138	A0A0D2T1A0	EC 3.1.4.4	phospholipase D [OS=Gossypium raimondii]	5	2	-0.4	0.38	0.6	0.24	0.015	-1.06	M	Lipid metabolism
139	A0A5C7I6U5	-	SKP1-like protein OS=Acer yangbiense OX=1000413 GN=EZV62_011742 PE=3 SV=1	23	2	-0.5	0.30	0.5	0.20	0.008	-1.02	M	Protein
140	A0A5B6YZY0	EC 1.6.5.2	NAD(P)H dehydrogenase (quinone) (Fragment) OS=Davidia involucrata OX=16924 GN=Din_006702 PE=3 SV=1	16	2	1.0	0.33	2.0	0.22	0.013	-0.99	M	Mitochondrial electron transport / ATP synthesis
141	A0A1S2YYS8	-	Soluble inorganic pyrophosphatase [OS=Cicer arietinum]	34	3	1.8	0.22	2.8	0.21	0.006	-0.96	M	Not assigned
142	A0A0M4BU78	-	phospholipid/glycerol acyltransferase [OS=Jatropha curcas]	7	2	-4.1	0.23	-3.2	0.48	0.048	-0.87	M	Lipid metabolism
143	A0A1S3XYU8	EC 7.1.1.8	cytochrome b5-like [OS=Nicotiana tabacum]	40	3	0.3	0.31	1.2	0.20	0.016	-0.85	M	Redox
144	A0A6A1W5Y5	-	Proteasome subunit alpha type OS=Morella rubra OX=262757 GN=CJ030_MR3G009388 PE=3 SV=1	22	4	2.6	0.27	3.4	0.11	0.008	-0.82	M	Protein
145	A0A445B002	-	PHB domain-containing protein OS=Arachis hypogaea OX=3818 GN=Ahy_B01g056985 PE=4 SV=1	26	2	0.8	0.35	1.6	0.25	0.031	-0.80	M	Not assigned
146	A0A103YM86	-	Ran GTPase [OS=Cynara cardunculus var. scolymus]	26	2	0.4	0.47	1.1	0.15	0.050	-0.79	M	Signalling
147	A0A0K9QZT7	-	Importin subunit alpha [OS=Spinacia oleracea]	14	2	2.1	0.20	2.8	0.16	0.008	-0.71	M	Protein

148	V5PZM8	-	Nascent polypeptide-associated complex subunit beta [OS=Suaeda glauca]	32	3	1.6	0.21	2.3	0.12	0.008	-0.70	M	Protein
149	A0A4Y7JEW5	-	NTF2 domain-containing protein OS=Papaver somniferum OX=3469 GN=C5167_006664 PE=4 SV=1	30	2	1.8	0.26	2.5	0.14	0.014	-0.70	M	Not assigned
150	A0A251V3Z2	-	Putative GTP-binding protein yptV4 OS=Helianthus annuus OX=4232 GN=YPTV4 PE=4 SV=1	27	4	1.1	0.07	1.7	0.17	0.004	-0.66	M	Not assigned
151	Q29Q34	-	At5g19440 [OS=Arabidopsis thaliana]	12	2	2.0	0.16	2.6	0.28	0.025	-0.66	M	Cell wall
152	A0A1W6AJY4	-	Heat shock protein 70 [OS=Camellia sinensis]	36	2	1.5	0.24	2.1	0.06	0.011	-0.65	M	Stress
153	A0A6B7K5R6	EC:1.2.1.8	Betaine aldehyde dehydrogenase OS=Hylocereus undatus OX=176265 GN=BADH PE=2 SV=1	18	3	2.4	0.22	3.1	0.21	0.027	-0.61	M	Amino acid metabolism
154	A0A328E8M1	-	TCTP domain-containing protein OS=Cuscuta australis OX=267555 GN=DM860_001472 PE=3 SV=1	26	2	3.2	0.16	3.8	0.09	0.005	-0.59	M	Not assigned
155	A0A6A4LQ78	EC 3.4.25.1	Proteasome endopeptidase complex (Fragment) OS=Rhododendron williamsianum OX=262921 GN=C3L33_05025 PE=4 SV=1	13	2	1.3	0.30	1.9	0.13	0.034	-0.59	M	Protein
156	A0A0K9R7U1	-	T-complex protein 1 subunit delta [OS=Spinacia oleracea]	25	2	3.4	0.16	4.0	0.08	0.005	-0.59	M	Protein
157	A0A059BCV0	-	coatomer subunit alpha [OS=Eucalyptus grandis]	10	2	1.1	0.16	1.6	0.10	0.005	-0.59	M	Cell
158	Q42434	-	Luminal-binding protein [OS=Spinacia oleracea]	21	3	3.9	0.31	3.9	0.09			U	Protein
159	A0A2G5CB18	-	Fn3_like domain-containing protein (Fragment) OS=Aquilegia coerulea OX=218851 GN=AQUCO_06900035v1 PE=3 SV=1	3	2	-1.4	1.11	-1.5	0.93			U	Not assigned
160	A0A200QLS2	EC 2.7.9.1	pyruvate, phosphate dikinase [OS=Macleaya cordata]	8	2	3.1	0.54	3.1	0.64			U	CHO metabolism
161	A0A199VR07	-	Pyrophosphate-energized vacuolar membrane proton pump [OS=Ananas comosus]	14	2	4.0	0.85	4.0	0.46			U	Transport
162	A0A0V0HH97	-	Putative ovule protein [OS=Solanum chacoense]	21	3	0.3	0.16	0.3	0.10			U	Not assigned
163	A0A067LBG3	EC 6.2.1.5	Succinate--CoA ligase [ADP-forming] subunit alpha, mitochondrial [OS=Jatropha curcas]	13	2	2.2	0.28	2.2	0.33			U	TCA / org transformation

164	A0A2G5EQ64	EC 4.2.1.3	Aconitate hydratase OS=Aquilegia coerulea OX=218851 GN=AQUCO_00500057v1 PE=3 SV=1	2	2	0.1	0.50	0.2	0.37		U	TCA / org transformation
165	A0A2C9WFY8	-	E1 ubiquitin-activating enzyme OS=Manihot esculenta OX=3983 GN=MANES_02G214500 PE=3 SV=1	7	2	0.2	0.34	0.1	0.52		U	Not assigned
166	S8CY20	-	prephenate aminotransferase [OS=Genlisea aurea]	7	2	-1.0	0.34	-1.1	0.16		U	Amino acid metabolism
167	A0A200R6C8	-	Isocitrate dehydrogenase [NAD] subunit, mitochondrial [OS=Macleaya cordata]	12	3	1.2	0.16	1.2	0.07		U	TCA / org transformation
168	A0A078H8Z2	-	BnaA01g32760D protein [OS=Brassica napus]	14	3	1.3	0.19	1.2	0.72		U	Not assigned
169	A0A2S1CJD3	[EC:4.1.1.1	Pyruvate decarboxylase OS=Hylocereus undatus OX=176265 GN=PDC PE=2 SV=1	10	3	0.4	0.76	0.3	0.11		U	Fermentation
170	A0A6A1VYJ7	-	Leuk-A4-hydro_C domain-containing protein OS=Morella rubra OX=262757 GN=CJ030_MR3G014568 PE=3 SV=1	7	2	-0.2	0.39	-0.1	0.26		U	Not assigned
171	A0A5B6ZEA2	EC 3.4.16.-	Carboxypeptidase OS=Davidia involucrata OX=16924 GN=Din_011662 PE=3 SV=1	9	2	-0.5	2.04	0.0	0.88		U	Protein
172	J7GHV7	EC 1.1.1.284	S-(hydroxymethyl)glutathione dehydrogenase [OS=Lactuca sativa]	28	3	2.1	0.21	2.3	0.56		U	Fermentation
173	Q8RU73	EC 5.3.1.6	Chloroplast ribose-5-phosphate isomerase [OS=Spinacia oleracea]	16	2	-2.8	2.33	-3.2	0.64		U	OPP
174	A2Q4Y9	EC 6.1.1.4	Aminoacyl-tRNA synthetase, class Ia [OS=Medicago truncatula]	2	2	0.2	0.17	0.2	0.15		U	Protein
175	A0A1R3GPW9	-	isochorismatase-like protein [OS=Corchorus capsularis]	12	2	0.0	0.42	0.2	0.55		U	Co-factor and vitamine metabolism
176	A0A090BCP6	-	methionine synthase [OS=Gentiana triflora]	18	3	-0.3	1.15	0.0	0.48		U	Amino acid metabolism

177	A0A6A4M1R3	-	GST N-terminal domain-containing protein (Fragment) OS=Rhododendron williamsianum OX=262921 GN=C3L33_02758 PE=4 SV=1	4	2	2.3	0.64	2.2	0.41		U	Not assigned
178	D8SW83	EC 3.3.1.1	Adenosylhomocysteinase [OS=Selaginella moellendorffii]	24	2	-1.8	0.47	-2.2	1.45		U	Redox
179	A0A1D6HXT2	-	Heteroglycan glucosidase 1 [OS=Zea mays]	2	2	-0.1	0.52	0.1	0.16		U	CHO metabolism
180	A0A1Y3BYV5	-	Putative ATP synthase YMF19-like, ATP synthase YMF19 [OS=Helianthus annuus]	9	2	1.6	0.41	1.4	0.23		U	Not assigned
181	A0A5N6QUJ3	-	Peptidase_M3 domain-containing protein OS=Carpinus fangiana OX=176857 GN=FH972_006834 PE=3 SV=1	2	2	-0.6	0.90	-0.3	0.42		U	Not assigned
182	A0A4V6AA51	EC:1.4.1.2	Glutamate dehydrogenase OS=Populus alba OX=43335 GN=D5086_0000118890 PE=3 SV=1	24	3	3.5	0.39	3.4	0.07		U	N-metabolism
183	A0A199UYE4	EC 1.8.1.4	Dihydrolipoyl dehydrogenase [OS=Ananas comosus]	16	2	0.9	0.14	0.8	0.08		U	Lipid metabolism
184	A0A0J8BEJ3	EC 3.4.19.12	ubiquitinyl hydrolase 1 [OS=Beta vulgaris subsp. vulgaris]	4	2	-0.9	0.87	-1.2	0.23		U	RNA
185	Q41386	-	pullulanase [OS=Spinacia oleracea]	3	2	-1.8	0.26	-1.6	0.64		U	Not assigned
186	B6U0Y9	-	ATP binding protein [OS=Zea mays]	5	2	-2.8	1.39	-2.2	0.87		U	Protein
187	A8JA18	-	ClpB chaperone, Hsp100 family [OS=Chlamydomonas reinhardtii]	3	2	1.6	0.92	2.0	0.20		U	Not assigned
188	A0A103XXE9	-	Armadillo-type fold [OS=Cynara cardunculus var. scolymus]	2	2	-3.1	1.16	-2.7	0.13		U	Transport
189	A0A0B0PXG2	EC 2.7.7.9	UTP--glucose-1-phosphate uridylyltransferase [OS=Gossypium arboreum]	17	2	-0.9	0.63	-0.7	0.08		U	Not assigned
190	A0A383VX59	EC 1.3.5.1	Succinate dehydrogenase [ubiquinone] flavoprotein subunit, mitochondrial OS=Tetradesmus obliquus OX=3088 GN=BQ4739_LOCUS10298 PE=3 SV=1	15	2	2.2	0.53	2.0	0.13		U	TCA / org transformation
191	A0A068VCD9	EC 3.1.3.16	Serine/threonine-protein phosphatase [OS=Coffea canephora]	35	5	1.7	0.11	1.3	1.03		U	Protein

192	A0A061END0	-	Ribosomal L22e protein family isoform 1 [OS=Theobroma cacao]	20	2	1.7	0.50	1.5	0.18	U	Protein
193	A0A200PNP8	-	Glycosyl transferase [OS=Macleaya cordata]	4	2	1.6	0.18	1.7	0.13	U	CHO metabolism
194	A0A251VBP5	-	Putative crotonase superfamily OS=Helianthus annuus OX=4232 GN=HannXRQ_Chr03g0089451 PE=3 SV=1	8	2	-1.4	0.44	-2.0	1.37	U	Amino acid metabolism
195	A0A1U8E248	-	probable lactoylglutathione lyase, chloroplastic isoform X1 [OS=Capsicum annuum]	14	2	2.7	0.26	2.1	1.33	U	Biodegradation of Xenobiotics
196	A0A328E279	-	RRM domain-containing protein OS=Cuscuta australis OX=267555 GN=DM860_011028 PE=4 SV=1	11	2	-1.3	0.30	-2.6	2.94	U	Not assigned
197	Q5QIS5	EC:1.11.1.11	Ascorbate peroxidase [OS=Rehmannia glutinosa]	21	2	3.1	0.36	3.3	0.05	U	Redox
198	Q59I53	-	Mitochondrial F1-ATPase gamma subunit [OS=Ipomoea nil]	21	3	2.3	0.68	2.0	0.13	U	Not assigned
199	A0A0K9PHS8	-	H(+)-transporting two-sector ATPase [OS=Zostera marina]	37	2	4.7	0.37	4.6	0.12	U	Not assigned
200	A0A5A7QFD9	-	Carboxymethylenebutenolidase OS=Striga asiatica OX=4170 GN=STAS_19932 PE=4 SV=1	11	2	2.2	0.23	1.7	1.09	U	Biodegradation of Xenobiotics
201	A0A0J8BDJ4	EC 2.7.7.9	Uncharacterized protein [OS=Beta vulgaris subsp. vulgaris]	12	3	3.7	0.32	3.9	0.03	U	Unclear classification
202	A0A1Z4EAX4	EC 1.1.1.95	D-3-phosphoglycerate dehydrogenase [OS=Beta vulgaris]	14	3	2.9	0.24	3.0	0.16	U	Amino acid metabolism
203	A0A6A4M4P8	-	Clathrin-link domain-containing protein (Fragment) OS=Rhododendron williamsianum OX=262921 GN=C3L33_02908 PE=3 SV=1	13	2	-0.4	0.72	-0.8	0.53	U	Protein
204	A0A6A4M1C4	-	WD_REPEATS_REGION domain-containing protein (Fragment) OS=Rhododendron williamsianum OX=262921 GN=C3L33_01014 PE=4 SV=1	16	2	2.4	0.32	2.5	0.24	U	Not assigned
205	A0A2C9WDJ4	EC 5.3.1.5	Xylose isomerase OS=Manihot esculenta OX=3983 GN=MANES_02G134800 PE=3 SV=1	8	2	0.3	0.31	-0.2	1.02	U	CHO metabolism
206	B9R816	EC 1.3.1.74	cysteine protease, putative [OS=Ricinus communis]	6	2	1.6	0.50	1.9	0.39	U	Protein

207	S8E9H9	-	malic enzyme [OS=Genlisea aurea]	9	3	1.9	1.14	2.5	0.08	U	TCA / org transformation
208	A0A2I4DDB9	-	phototropin-1-like isoform X1 OS=Juglans regia OX=51240 GN=LOC108979031 PE=4 SV=1	6	2	0.5	1.17	1.1	0.24	U	Protein
209	A0A5N6R2W1	-	CBM20 domain-containing protein OS=Carpinus fangiana OX=176857 GN=FH972_008685 PE=3 SV=1	5	2	1.1	0.29	0.2	1.74	U	Not assigned
210	A0A061GHV7	-	Arabinose kinase isoform 1 [OS=Theobroma cacao]	3	2	-5.1	0.80	-4.6	0.73	U	Cell wall
211	A0A1U8APZ2	-	Probable plastid-lipid-associated protein 6, chloroplastic [OS=Nelumbo nucifera]	9	2	3.0	1.27	2.4	0.15	U	Cell
212	A0A445G1M6	-	Polyubiquitin-A OS=Glycine soja OX=3848 GN=D0Y65_044839 PE=4 SV=1	36	3	2.4	0.74	2.8	0.43	U	Protein
213	A0A2R6R2L5	-	D-fructose-1,6-bisphosphate 1-phosphohydrolase OS=Actinidia chinensis var. chinensis OX=1590841 GN=CEY00_Acc11580 PE=3 SV=1	16	2	1.2	1.16	0.6	0.14	U	Glycolysis
214	Q94CF7	-	Cytosolic ascorbate peroxidase [OS=Suaeda salsa]	15	2	3.1	0.56	3.5	0.25	U	Redox
215	A0A1U8A2K9	-	nuclear transcription factor Y subunit C-9-like [OS=Nelumbo nucifera]	13	3	-0.3	0.94	0.2	0.24	U	RNA
216	A0A072TFZ8	-	COP9 signalosome complex subunit 4 OS=Medicago truncatula OX=3880 GN=MTR_0173s0030 PE=3 SV=1	12	2	-5.2	0.85	-3.8	2.24	U	Signalling
217	A0A1R3GZU8	-	sulfurtransferase [OS=Corchorus olitorius]	15	3	2.6	0.10	1.8	1.35	U	Development
218	A0A1U8A3I7	-	Probable mitochondrial-processing peptidase subunit beta, mitochondrial [OS=Nelumbo nucifera]	6	2	1.3	0.73	1.8	0.15	U	Not assigned
219	A0A1R3I8W3	-	Ureohydrolase [OS=Corchorus olitorius]	18	2	1.9	0.93	2.4	0.05	U	Amino acid metabolism
220	A0A5J4ZPP9	-	HATPase_c domain-containing protein OS=Nyssa sinensis OX=561372 GN=F0562_011536 PE=3 SV=1	29	3	1.1	1.89	2.3	0.11	U	Not assigned
221	W0FHL5	EC 1.11.1.6	catalase [OS=Hylocereus undatus]	43	10	7.6	0.45	7.3	0.18	U	Redox

222	A0A3G4R8P9	-	Phenylcoumaran benzylic ether reductase-like protein I (Fragment) OS=Ferocactus pilosus OX=867057 PE=2 SV=1	23	2	2.2	0.74	2.7	0.38	U	Not assigned
223	A0A199UXZ2	EC:1.1.99.21	Sorbitol dehydrogenase [OS=Ananas comosus]	15	2	-0.9	0.67	-2.2	1.84	U	Not assigned
224	A0A0K9R992	EC 1.1.1.205	inosine-5'-monophosphate dehydrogenase [OS=Spinacia oleracea]	7	2	-0.9	0.55	-1.3	0.11	U	Nucleotide metabolism
225	M4FA64	EC 6.3.5.1	glutamine-dependent NAD(+) synthetase [OS=Brassica rapa subsp. pekinensis]	4	2	-3.6	0.27	-3.3	0.39	U	Amino acid metabolism
226	A0A498K093	-	UBX domain-containing protein OS=Malus domestica OX=3750 GN=DVH24_001265 PE=4 SV=1	2	2	-2.1	1.12	-1.3	0.32	U	Protein
227	A0A1S3CJ54	-	reactive Intermediate Deaminase A, chloroplastic-like [OS=Cucumis melo]	20	3	0.5	1.06	1.4	0.60	U	Not assigned
228	A0A0K9RZ84	EC 3.4.19.12	Uncharacterized protein [OS=Spinacia oleracea]	5	2	-0.9	0.63	-0.5	0.14	U	Unclear classification
229	A0A1E5ULN3	-	calnexin-like protein [OS=Dichanthelium oligosanthes]	5	2	0.7	0.49	0.1	0.58	U	Signalling
230	A0A445EN18	-	CYTOSOL_AP domain-containing protein OS=Arachis hypogaea OX=3818 GN=Ahy_A01g001368 PE=3 SV=1	14	2	2.9	0.23	2.4	0.69	U	Not assigned
231	A0A1L5JKC2	-	tubulin alpha chain [OS=Sesuvium portulacastrum]	51	4	4.5	0.24	4.8	0.48	U	Cell
232	A0A0J8BCR7	EC 6.3.2.2	Uncharacterized protein [OS=Beta vulgaris subsp. vulgaris]	10	2	-0.3	0.78	-0.9	0.25	U	Unclear classification
233	A0A097PL31	-	Nitrilase/cyanide hydratase and apolipoprotein N-acyltransferase family protein [OS=Acorus calamus]	18	2	0.5	0.06	0.1	0.55	U	Muscellaneous
234	A0A1U8MJ75	-	cinnamyl alcohol dehydrogenase 1-like [OS=Gossypium hirsutum]	3	2	-3.0	0.77	-4.1	1.29	U	Secondary metabolism
235	A0A498HZW4	-	AICAR transformylase OS=Malus domestica OX=3750 GN=DVH24_019258 PE=3 SV=1	3	2	-0.6	0.47	-2.0	1.82	U	Not assigned

236	A0A0B0PEQ9	-	flavoprotein wrbA [OS=Gossypium arboreum]	13	2	0.7	0.40	1.4	0.83	U	Mitochondrial electron transport / ATP synthesis
237	A0A5J5AR11	-	PKS_ER domain-containing protein OS=Nyssa sinensis OX=561372 GN=F0562_006186 PE=3 SV=1	11	2	2.3	0.43	1.6	0.85	U	Not assigned
238	A0A2U1QJ36	EC 2.5.1.6	S-adenosylmethionine synthase OS=Artemisia annua OX=35608 GN=CTI12_AA023940 PE=3 SV=1	15	2	-1.9	2.00	-0.3	0.14	U	Amino acid metabolism
239	A0A2H9ZWF0	EC 2.2.1.2	Transaldolase OS=Apostasia shenzhenica OX=1088818 GN=AXF42_Ash014788 PE=3 SV=1	10	2	0.7	0.32	1.2	0.50	U	OPP
240	A0A1D1ZLC3	-	Chlorophyll a-b binding protein, chloroplastic [OS=Anthurium amnicola]	14	2	1.4	0.93	2.5	1.16	U	Photosynthesis
241	T2DNI2	EC 1.3.1.33	NADPH-protochlorophyllide oxidoreductase [OS=Phaseolus vulgaris]	7	2	-0.4	0.43	0.0	0.28	U	Secondary metabolism
242	A0A103YJ12	EC 1.16.3.1	ferritin [OS=Cynara cardunculus var. scolymus]	7	2	0.8	0.33	1.2	0.30	U	Nucleotide metabolism
243	A0A151T4Z0	-	Putative syntaxin-131 family [OS=Cajanus cajan]	8	2	-0.9	0.18	-1.9	1.19	U	Cell
244	A0A4D8YSA4	-	Ubiquinol-cytochrome c reductase cytochrome c1 subunit OS=Salvia splendens OX=180675 GN=CYC1 PE=4 SV=1	12	2	1.0	0.20	-1.3	2.80	U	Protein
245	A0A438FGX7	-	Histidine--tRNA ligase OS=Vitis vinifera OX=29760 GN=VvCHDh000037_1 PE=3 SV=1	8	3	0.2	0.28	-0.4	0.61	U	Amino acid metabolism
246	A5BCM7	-	Mitochondrial pyruvate carrier [OS=Vitis vinifera]	8	2	1.4	0.41	1.1	0.08	U	Transport
247	A0A2C9U1Z4	-	PEROXIDASE_4 domain-containing protein OS=Manihot esculenta OX=3983 GN=MANES_18G064300 PE=3 SV=1	11	2	-0.7	0.43	-1.3	0.46	U	Not assigned
248	A0A200RA69	-	Isocitrate and isopropylmalate dehydrogenases family [OS=Macleaya cordata]	12	4	1.7	0.21	1.9	0.04	U	Not assigned
249	Q9AVF9	-	porphobilinogen deaminase [OS=Amaranthus tricolor]	14	2	-1.0	0.48	-1.5	0.41	U	Secondary metabolism

250	A0A200QKR7	-	TATA-binding protein interacting (TIP20) [OS=Macleaya cordata]	5	3	0.2	0.55	-0.4	0.34		U	Protein
251	A0A1J3CUJ5	-	Thiamine thiazole synthase, chloroplastic [OS=Noccaea caerulescens]	9	2	1.0	0.33	0.5	0.50		U	Co-factor and vitamine metabolism
252	A0A6B7K6I3	-	Stem-specific protein OS=Hylocereus undatus OX=176265 GN=TSJT1 PE=2 SV=1	18	3	-0.1	0.33	-1.7	1.65		U	Not assigned
253	A0A1J7H2T7	EC 2.5.1.19	3-phosphoshikimate 1-carboxyvinyltransferase [OS=Lupinus angustifolius]	9	3	-2.6	1.63	-4.1	0.45		U	Secondary metabolism
254	A0A5A7QKD0	-	Citrulline--aspartate ligase OS=Striga asiatica OX=4170 GN=STAS_21252 PE=3 SV=1	7	7	1.8	0.47	2.2	0.07		U	Amino acid metabolism
255	A0A2H3YQT8	EC 3.1.3.2	Purple acid phosphatase OS=Phoenix dactylifera OX=42345 GN=LOC103716165 PE=3 SV=1	4	2	-1.4	2.10	0.6	0.63		U	Miscellaneous
256	A0A249Y714	EC 5.1.3.-	UDP-glucose 4-epimerase OS=Hylocereus polyrhizus OX=1195597 GN=UGE1 PE=2 SV=1	13	3	-1.5	0.60	-0.8	0.36		U	Not assigned
257	A0A1D6KRE0	-	Membrane steroid-binding protein 1 [OS=Zea mays]	6	2	0.8	0.47	1.3	0.13		U	Not assigned
258	Q9AVU8	EC 3.6.1.34	Putative vacuolar ATP Synthase subunit A [OS=Mesembryanthemum crystallinum]	40	3	4.3	0.80	3.4	0.43		U	Transport
259	A0A2I4E570	EC:1.2.4.2	2-oxoglutarate dehydrogenase, mitochondrial OS=Juglans regia OX=51240 GN=LOC108986378 PE=3 SV=1	9	2	-0.4	0.31	-1.4	1.08		U	TCA / org transformation.
260	A0A1S2Z641	-	Monocopper oxidase-like protein SKU5 [OS=Cicer arietinum]	5	2	0.5	0.34	0.0	0.39		U	Not assigned
261	A0A200Q746	-	guanosine nucleotide diphosphate dissociation inhibitor [OS=Macleaya cordata]	21	3	2.7	0.14	3.1	0.33		U	Signalling
262	A0A5N6QR13	EC:1.11.1.11	L-ascorbate peroxidase OS=Carpinus fangiana OX=176857 GN=FH972_005233 PE=3 SV=1	23	2	2.3	0.49	2.9	0.34		U	Redox
263	V4SRJ0	EC 1.11.1.15	Uncharacterized protein [OS=Citrus clementina]	14	3	5.3	0.75	4.5	0.17		U	Unclear classification

264	A0A445A1Y9	-	J domain-containing protein OS=Arachis hypogaea OX=3818 GN=Ahy_B03g065578 PE=4 SV=1	1	2	-3.8	0.84	-3.0	0.20	U	Cell
265	A0A0K9QSS0	EC 3.2.1.26	Uncharacterized protein [OS=Spinacia oleracea]	5	2	-1.3	0.74	-0.6	0.15	U	Unclear classification
266	A0A2J6K3D6	-	Transket_pyr domain-containing protein OS=Lactuca sativa OX=4236 GN=LSAT_8X58500 PE=3 SV=1	6	2	-2.5	0.90	-1.6	0.16	U	Amino acid metabolism
267	A0A1Q3AUJ0	-	Kinesin domain-containing protein [OS=Cephalotus follicularis]	3	2	-1.7	0.40	-2.2	0.35	U	Cell
268	W9RTU4	EC:6.3.4.3	formate--tetrahydrofolate ligase [OS=Morus notabilis]	11	3	2.1	0.48	1.6	0.12	U	Not assigned
269	A0A1U8B872	-	aspartate--tRNA ligase 2, cytoplasmic-like [OS=Nelumbo nucifera]	4	2	4.6	0.31	3.9	0.57	U	Protein
270	A0A5N6MD63	-	Bac_surface_Ag domain-containing protein OS=Mikania micrantha OX=192012 GN=E3N88_34055 PE=4 SV=1	5	2	-0.2	0.32	-0.7	0.29	U	Not assigned
271	A0A0D2T673	EC 5.1.3.-	Uncharacterized protein [OS=Gossypium raimondii]	12	2	-1.5	0.73	-0.5	0.46	U	Unclear classification
272	A0A1S4BSA2	-	oxygen-evolving enhancer protein 1, chloroplastic-like [OS=Nicotiana tabacum]	24	3	6.5	0.53	5.9	0.10	U	Photosynthesis
273	A4LA97	-	chloroplast small heat shock protein [OS=Epilobium amurense]	9	2	4.3	0.95	3.1	0.43	U	Stress
274	A0A0E3Z7B6	-	dehydrin-like protein [OS=Leuchtenbergia principis]	33	2	-0.9	0.66	0.3	0.80	U	Stress
275	A0A5B7C1D9	EC 2.1.1.10	Putative homocysteine S-methyltransferase 3 (Fragment) OS=Davidia involucrata OX=16924 GN=Din_043935 PE=4 SV=1	12	2	0.8	0.96	1.9	0.13	U	Amino acid metabolism
276	A0A2U1QAH2	-	Class I glutamine amidotransferase-like superfamily protein OS=Artemisia annua OX=35608 GN=CTI12_AA054320 PE=4 SV=1	11	2	0.2	0.68	1.0	0.25	U	Not assigned
277	A0A1J3E1D9	-	Ubiquitin receptor RAD23c [OS=Noccaea caerulescens]	10	2	1.8	0.71	2.7	0.29	U	Protein

278	A0A1Q3BWE7	-	ABC_tran domain-containing protein/ABC_membrane domain-containing protein [OS=Cephalotus follicularis]	4	3	-1.4	0.90	-0.4	0.03		U	Protein
279	A0A1J3CV76	-	putative oxidoreductase, chloroplastic [OS=Noccaea caerulescens]	18	2	1.5	1.20	0.0	0.33		U	Redox
280	A0A1S3CQ00	EC:4.2.1.33; 4.2.1.35	3-isopropylmalate dehydratase large subunit [OS=Cucumis melo]	5	2	-0.7	0.32	-0.3	0.21		U	Amino acid metabolism
281	F2DJ31	EC 6.3.5.4	predicted protein [OS=Hordeum vulgare subsp. vulgare]	6	2	-2.4	0.51	-1.6	0.43		U	Not assigned
282	A0A4P1QW91	EC 2.7.1.90	Pyrophosphate--fructose 6-phosphate 1-phosphotransferase subunit beta OS=Lupinus angustifolius OX=3871 GN=PFP-BETA PE=3 SV=1	16	2	2.6	0.41	1.8	0.53		U	Glycolysis
283	A0A6A2XNF7	-	Glutamate--tRNA ligase OS=Hibiscus syriacus OX=106335 GN=F3Y22_tig00116961pilonHSYRG00170 PE=3 SV=1	6	2	-2.0	0.08	-2.4	0.36		U	Amino acid metabolism
284	B1NJ24	-	Delta 1-pyrroline-5-carboxylate synthetase [OS=Opuntia streptacantha]	7	2	-4.3	0.54	-3.5	0.39		U	Amino acid metabolism
285	Q9LRI8	EC 1.3.3.4	protoporphyrinogen oxidase [OS=Spinacia oleracea]	6	2	-0.8	0.33	-2.8	1.54		U	Secondary metabolism
286	A0A4Y7JLR9	EC:2.7.4.	Adenylate kinase OS=Papaver somniferum OX=3469 GN=C5167_022398 PE=3 SV=1	32	2	3.4	0.27	3.0	0.13		U	Nucleotide metabolism
287	Q45W80	EC 2.7.4.6	Nucleoside diphosphate kinase [OS=Arachis hypogaea]	27	4	5.0	0.58	5.7	0.10		U	Nucleotide metabolism
288	A0A0B5KU79	EC 2.4.1.18	Starch branching enzyme [OS=Lilium davidii var. unicolor]	4	2	1.2	0.41	-0.3	1.12		U	CHO metabolism
289	Q8VYF5-2	-	Isoform 2 of N-carbamoylputrescine amidase [OS=Arabidopsis thaliana]	27	3	-2.1	0.43	-1.4	0.19		U	Not assigned
290	A0A0K9PFE3	EC 7.1.2.1	plasma membrane ATPase [OS=Zostera marina]	11	2	0.1	1.18	-2.6	1.53		U	Not assigned
291	A0A1U8MAX5	-	ADP,ATP carrier protein 3, mitochondrial-like [OS=Gossypium hirsutum]	18	2	5.8	0.27	5.4	0.09		U	Transport

292	A0A199W759	-	Villin-4 [OS=Ananas comosus]	4	2	-3.0	0.17	-1.5	1.07	U	Cell
293	A0A1D1XWV8	-	putative aldehyde dehydrogenase [OS=Anthurium amnicola]	3	2	-1.5	0.49	-0.8	0.03	U	Fermentation
294	A0A1J3F7U6	-	Hsp70 nucleotide exchange factor FES1 [OS=Noccaea caerulescens]	6	2	0.2	0.41	0.8	0.04	U	Protein
295	A0A4S4DTM7	-	CCT-theta OS=Camellia sinensis var. sinensis OX=542762 GN=TEA_013292 PE=3 SV=1	14	3	2.5	0.55	3.3	0.11	U	Protein
296	A0A0K9S004	EC 4.2.1.24	Delta-aminolevulinic acid dehydratase [OS=Spinacia oleracea]	16	2	0.7	0.32	-1.3	1.30	U	Metabolism of cofactors and vitamins
297	A0A1J6HX35	-	Methionine--tRNA ligase, cytoplasmic [OS=Nicotiana attenuata]	4	2	-1.0	0.35	-0.3	0.26	U	Protein
298	T1RU75	EC 3.6.4.6	N-ethylmaleimide sensitive fusion protein [OS=Silene vulgaris]	8	4	-0.5	0.48	0.3	0.23	U	Protein
299	A0A5N5LRF6	-	Gp_dh_N domain-containing protein OS=Salix brachista OX=2182728 GN=DKX38_013310 PE=3 SV=1	13	2	-2.2	1.46	-4.4	0.34	U	Not assigned
300	A0A4S4E324	-	CS domain-containing protein OS=Camellia sinensis var. sinensis OX=542762 GN=TEA_003650 PE=4 SV=1	7	2	1.3	0.57	0.2	0.53	U	Not assigned
301	A0A1S4CD72	EC:1.8.1.7	glutathione reductase, chloroplastic isoform X1 [OS=Nicotiana tabacum]	4	2	0.8	0.24	-0.1	0.59	U	Redox
302	A0A2H3YSQ0	-	fasciclin-like arabinogalactan protein 8 OS=Phoenix dactylifera OX=42345 GN=LOC103716887 PE=3 SV=1	6	2	4.0	0.69	2.5	0.75	U	Not assigned
303	A5BGC9	EC 1.1.1.44	6-phosphogluconate dehydrogenase, decarboxylating [OS=Vitis vinifera]	15	2	-1.1	0.72	0.4	0.65	U	lipid metabolism
304	M8ANM1	EC:1.1.5.3	Glycerol-3-phosphate dehydrogenase SDP6, mitochondrial [OS=Aegilops tauschii]	2	2	-1.9	0.23	-3.4	0.95	U	Lipid metabolism

305	A8JJQ6	-	Histone H2B (Fragment) OS=Chlamydomonas reinhardtii OX=3055 GN=HBV2 PE=3 SV=1	10	2	3.6	0.64	2.3	0.61	U	DNA
306	A0A446L2X6	-	UVR domain-containing protein OS=Triticum turgidum subsp. durum OX=4567 GN=TRITD_2Av1G174080 PE=3 SV=1	19	2	0.2	0.75	-1.0	0.20	U	DNA
307	G5DXH2	-	Cullin-associated NEDD8-dissociated protein [OS=Silene latifolia]	9	2	0.4	0.25	-0.1	0.14	U	Hormone metabolism
308	A0A1U7WW63	-	Kynurenine--oxoglutarate transaminase [OS=Nicotiana sylvestris]	8	2	-0.2	0.20	-0.6	0.16	U	Amino acid metabolism
309	A0A1U8JHS1	EC:4.2.3.1	threonine synthase 1, chloroplastic-like [OS=Gossypium hirsutum]	7	2	-2.8	0.12	-3.2	0.21	U	Amino acid metabolism
310	A0A6B7K9X7	EC:2.6.1.1	Aspartate aminotransferase OS=Hylocereus undatus OX=176265 GN=GOT1 PE=2 SV=1	40	9	5.3	0.16	5.0	0.14	U	Amino acid metabolism
311	A0A4D8YFJ8	EC:6.3.5.5	Carbamoyl-phosphate synthase (glutamine-hydrolyzing) OS=Salvia splendens OX=180675 GN=carB PE=3 SV=1	5	2	-0.5	0.07	-0.9	0.08	U	Nucleotide metabolism
312	A0A200QY87	-	semialdehyde dehydrogenase [OS=Macleaya cordata]	10	3	-0.2	0.16	-0.6	0.09	U	Amino acid metabolism
313	B9HB41	-	MIF4G domain-containing family protein [OS=Populus trichocarpa]	3	2	-0.6	0.04	-0.7	0.05	U	RNA
314	O81340	-	26S proteasome regulatory subunit S5A [OS=Mesembryanthemum crystallinum]	14	3	1.9	0.13	2.3	0.07	U	Protein
315	A0A0B2PPC9	EC 1.6.-.-	putative quinone-oxidoreductase like, chloroplastic [OS=Glycine soja]	17	4	1.7	0.08	2.1	0.12	U	Not assigned
316	A0A061GXS5	-	OSBP(Oxysterol binding protein)-related protein 3C isoform 2 [OS=Theobroma cacao]	6	2	-1.2	0.19	-0.7	0.11	U	Cell
317	A0A097PLX8	-	LOS4 [OS=Pinellia ternata]	9	2	0.7	0.31	1.2	0.03	U	Not assigned

318	A0A061GUW9	-	Nucleotidylyl transferase superfamily protein isoform 2 [OS=Theobroma cacao]	10	3	-0.2	0.14	0.3	0.07		U	Co-factor and vitamine metabolism
319	A0A078GBB1	-	BnaC08g42630D protein [OS=Brassica napus]	7	2	2.4	0.25	3.0	0.15		U	Not assigned

a Accession number, according to Uniprot database

b Coverage.

c M.P., number of matched unique peptide. The proteins with ≥ 2 matched peptides were considered.

d Protein abundance. Values of protein abundance are logarithmically transformed and are the mean of triplicates.

e Difference in protein abundance. Differences are obtained by subtraction (Exocarp - Mesocarp).

f Distribution between tissues. Proteins assigned a fold change cutoff at 1.5 (> |0.5849|) for difference were considered to be differentially distributed (*p*<0.05).

e, E, significantly abundant in exocarp; M, significantly abundant in mesocarp; U, Uniformly distributed.

g Function, functional bin code using classification by Mapman bin code.

Supplementary Table 3. List of affected proteins by salt stress

	Accession[a]	Description	Functional classification[b]	Coverage (%)	M.P.[c]	0 mM		250 mM		p-value	Difference[e]
						Abundance[d]	S.D.	Abundance[d]	S.D.		
1	M8BQ11	Enoyl-[acyl-carrier-protein] reductase [NADH], chloroplastic [OS=Aegilops tauschii]	Unclear classification	13	2	-4.3	1.16	-2.0	0.58	0.038	2.28
2	A0A075J5M8	phosphoenolpyruvate carboxylase [OS=Opuntia cochenillifera]	Glycolysis	56	18	6.9	0.58	8.5	0.28	0.014	1.56
3	A0A199W0F4	ADP,ATP carrier protein [OS=Ananas comosus]	Transport	5	2	-2.9	0.10	-1.6	0.09	0.000	1.30
4	A0A1U7ZBG4	CSC1-like protein ERD4 [OS=Nelumbo nucifera]	Not assigned	3	2	1.7	0.11	2.7	0.31	0.006	1.01
5	A0A1U7YSG9	pheophorbide a oxygenase, chloroplastic-like [OS=Nelumbo nucifera]	Tetrapyrrole synthesis	6	2	-0.8	0.07	0.0	0.20	0.003	0.81
6	F2DAY3	lactoylglutathione lyase [OS=Hordeum vulgare subsp. vulgare]	Biodegradation of Xenobiotics	10	2	0.9	0.18	1.7	0.31	0.021	0.75
7	A0A0B0P8D2	ATP synthase subunit gamma, mitochondrial [OS=Gossypium arboreum]	Photosynthesis	12	2	-1.1	0.18	-0.3	0.42	0.047	0.75
8	M5X090	cysteine synthase [OS=Prunus persica]	Amino acid metabolism	7	2	0.4	0.27	1.2	0.17	0.015	0.73
9	M0RYE9	glucose-1-phosphate adenylyltransferase [OS=Musa acuminata subsp. malaccensis]	Cell wall	18	2	1.2	0.24	1.9	0.23	0.020	0.72
10	A0A191T655	Photosystem I iron-sulfur center [OS=Cylindrocystis brebissonii]	Photosynthesis	64	5	5.2	0.15	5.9	0.26	0.019	0.65
11	A0A1U8AJJ9	fructose-1,6-bisphosphatase, chloroplastic-like [OS=Nelumbo nucifera]	Photosynthesis	14	3	4.3	0.31	4.9	0.17	0.036	0.63
12	F4YFD1	Alcohol dehydrogenase [OS=Camellia sinensis]	Fermentation	10	2	1.2	0.08	1.8	0.11	0.001	0.63
13	A0A1U8B3L9	Peroxiredoxin Q, chloroplastic [OS=Nelumbo nucifera]	Redox	24	3	3.8	0.32	4.5	0.13	0.038	0.61
14	T2C628	phospholipase D [OS=Brassica napus]	Lipid metabolism	7	2	2.8	0.07	3.4	0.13	0.002	0.59

15	A0A1R3GLZ3	ATPase, AAA-type, core [OS=Corchorus capsularis]	Transport	30	2	5.8	0.28	5.2	0.12	0.029	-0.59
16	A0A1J3E1D9	Ubiquitin receptor RAD23c [OS=Noccaea caerulescens]	Protein	10	3	1.1	0.24	0.5	0.04	0.009	-0.66
17	A0A1U8I3L7	actin-7-like [OS=Gossypium hirsutum]	Cell	56	2	6.3	0.18	5.7	0.13	0.006	-0.67
18	P42824	DnaJ protein homolog 2 [OS=Allium ampeloprasum]	Cell	9	2	0.3	0.22	-0.4	0.28	0.026	-0.71
19	A0A1U7Z672	hypersensitive-induced response protein 1-like isoform X1 [OS=Nelumbo nucifera]	Unclear classification	14	3	2.2	0.16	1.5	0.35	0.032	-0.71
20	A0A0K9RWJ9	Clathrin heavy chain [OS=Spinacia oleracea]	Cell	16	2	1.2	0.46	0.5	0.08	0.050	-0.75
21	W9SDS9	coatomer subunit alpha [OS=Morus notabilis]	Unclear classification	4	2	-2.1	0.24	-2.9	0.31	0.023	-0.82
22	A0A1Q3B0T2	Ribosomal_L22 domain-containing protein [OS=Cephalotus follicularis]	Unclear classification	16	2	1.8	0.20	1.0	0.31	0.018	-0.82
23	X5I186	glyceraldehyde-3-phosphate dehydrogenase [OS=Ipomoea nil]	Photosynthesis	24	2	5.2	0.04	4.3	0.47	0.030	-0.90
24	A0A0E3KJY5	Heat shock protein 70 [OS=Bryum argenteum]	Stress	32	2	1.5	0.49	0.6	0.19	0.039	-0.91
25	A0A0J8D3U4	Clustered mitochondria protein homolog OS=Beta vulgaris subsp. vulgaris GN=BVRB_2g029530 PE=3 SV=1	Unclear classification	2	2	-2.2	0.45	-3.1	0.22	0.031	-0.94
26	A0A059BK11	Polyadenylate-binding protein [OS=Eucalyptus grandis]	RNA	8	2	1.4	0.20	0.5	0.36	0.016	-0.94
27	A0A1D6QAN9	ATG8-interacting protein 1 [OS=Zea mays]	Unclear classification	5	2	-0.1	0.12	-1.1	0.07	0.000	-1.05
28	A0A0K9S0T3	Eukaryotic translation initiation factor 3 subunit C [OS=Spinacia oleracea]	Protein	4	2	0.3	0.23	-0.7	0.11	0.002	-1.07
29	W0FHL5	catalase [OS=Hylocereus undatus]	Redox	44	12	8.0	0.23	6.8	0.18	0.002	-1.17
30	A0A0C9RUD3	Serine/threonine-protein phosphatase [OS=Wollemia nobilis]	RNA	14	2	-0.8	0.11	-1.9	0.52	0.019	-1.17
31	A0A0B2SQP6	60S ribosomal protein L6 [OS=Glycine soja]	Protein	17	2	2.4	0.09	1.2	0.38	0.007	-1.18
32	A0A1S2Z641	Monocopper oxidase-like protein SKU5 [OS=Cicer arietinum]	Miscellaneous	5	2	0.9	0.12	-0.3	0.69	0.037	-1.25

33	Q9LNE3	probable fructokinase-2 [OS=Arabidopsis thaliana]	Glycolysis	16	3	2.2	0.40	0.9	0.27	0.009	-1.31
34	B9GNT1	D-3-phosphoglycerate dehydrogenase [OS=Populus trichocarpa]	Amino acid metabolism	10	2	-1.0	0.59	-2.3	0.42	0.033	-1.33
35	A0A1U8JW28	pto-interacting protein 1-like isoform X1 [OS=Gossypium hirsutum]	Unclear classification	10	2	-0.8	0.20	-2.1	0.72	0.035	-1.35
36	A0A0D2KIA7	Ribulose bisphosphate carboxylase/oxygenaseactivase [OS=Monoraphidium neglectum]	Photosynthesis	14	2	-0.6	0.17	-2.1	0.42	0.005	-1.49
37	A0A1S3CDA7	Beta-galactosidase [OS=Cucumis melo]	Miscellaneous	4	2	-1.2	0.20	-3.2	0.09	0.000	-1.93
38	A0A0K9QML8	peroxidase [OS=Spinacia oleracea]	Miscellaneous	10	2	-0.9	1.22	-3.5	0.64	0.033	-2.54
39	C9EIN8	putative NADH dehydrogenase [OS=Olea europaea]	Mitochindrial Electron transport /ATP synthesis	21	2	-0.8	0.15	-3.7	0.32	0.000	-2.92

a Accession number, according to Uniprot database

b Function, functional bin code using classification by Mapman bin code.

c M.P., number of matched unique peptide. The proteins with ≥ 2 matched peptides were considered.

d Protein abundance. Values of protein abundance are logarithmically transformed and are the mean of triplicates.

e Difference in protein abundance. Differences are obtained by subtraction (Salt stress - control). Proteins assigned a fold change cutoff at 1.5 (> |0.5849|) for difference were used (p<0.05).

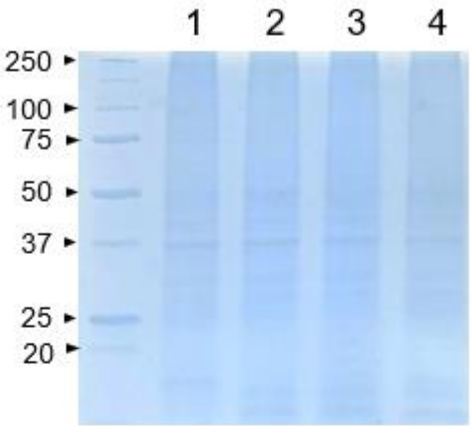

Supplementary Fig. 1. SDS-PAGE gel of exocarp proteins for evaluation of reprodicibility. Four technical replicates were prepared from one cladode. Proteins (5 µg for each sample) were visualized with Coomassie Brilliant Blue stain.
